# Commonality of Mechanism in Glycoside Hydrolases, Nucleoside Hydrolases, and Phosphorylases: Importance of Side-Chain Conformation Preorganization

**DOI:** 10.1021/jacsau.5c01069

**Published:** 2025-11-07

**Authors:** Po-Sen Tseng, Jonathan C. K. Quirke, W. Jonathan Lin, David Crich

**Affiliations:** † Department of Pharmaceutical and Biomedical Sciences, 1355 University of Georgia, 250 West Green Street, Athens, Georgia 30602, United States; ‡ Department of Chemistry, University of Georgia, 302 East Campus Road, Athens, Georgia 30602, United States; § Complex Carbohydrate Research Center, University of Georgia, 315 Riverbend Road, Athens, Georgia 30602, United States

**Keywords:** glycoside hydrolases, nucleoside hydrolases, nucleoside phosphorylases, electrostatic transition-state stabilization, substrate preorganization

## Abstract

A survey of the Protein Data Bank reveals that the arabinofuranosidase class of enzymes broadly restrict their substrate side chains to the *gauche,gauche* (*gg*) conformation that provides maximum electrostatic stabilization to oxocarbenium ion-like transition states and so employ the strategy reported previously for the majority of glycoside hydrolases, transglycosidases, and glycosyltransferases acting on pyranosyl substrates. The fructofuranosidases, ribonucleosidases, ribonucleoside phosphorylases, and nucleoside 2′-deoxyribosyltransferases, whose *gg* conformation is sterically hindered, restrict their substrate side chains to the next most positive charge-stabilizing *gauche,trans* (*gt*) conformation. These conclusions are supported by extensive literature studies on the mechanisms of C–N bond cleavage by members of the nucleosidase and nucleoside phosphorylase families and are discussed in terms of Warshel’s concept of the electrostatic origin of the catalytic power of enzymes and the role of preorganized active sites.

## Graphical abstract

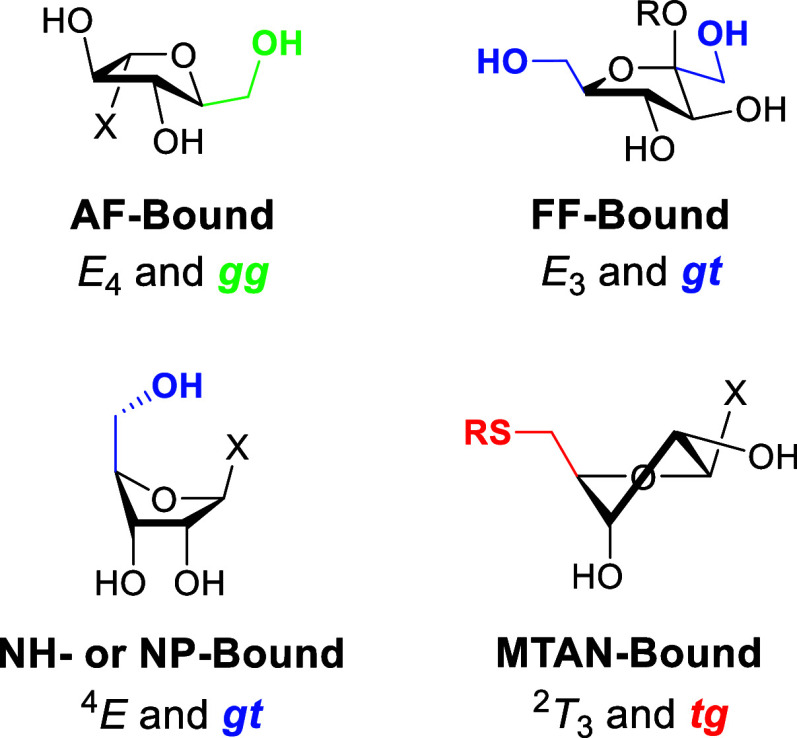

## Introduction

Glycoside hydrolases (GHs) and glycosyltransferases (GTs), enzymes that catalyze the hydrolysis or formation of glycosidic bonds with exceptional efficiency,^1^ with limited exceptions,^2–4^ function by stabilizing transition states with significant degrees of oxocarbenium ion character irrespective of their classifications as inverting or retaining (double inverting) systems.^5–9^ Optimal chemical glycosylation reactions are similarly best understood as proceeding via S_N_2-like mechanisms with exploded transition states with considerable oxocarbenium ion character.^9–12^ In chemical glycosylation or glycoside hydrolysis at the anomeric center of pyranoses, it has been established that the side-chain conformation influences both reactivity and stereoselectivity,^11,13–17^ with the *gauche,gauche* (*gg*) conformation providing maximal electrostatic stabilization to nascent positive charge at the oxocarbenium ion-like transition state. This influence of the side-chain conformation on reactivity at the anomeric center is analogous to the manner by which axial and pseudoaxial C–O bonds on pyranosyl rings enhance reactivity compared to the corresponding electron-withdrawing equatorial or pseudoequatorial bonds (Figure 1).^18–22^ Mining of the Protein Data Bank (PDB) revealed that GHs proceeding via classical mechanisms, Leloir GTs, and transglycosidases use hydrogen bonding to restrict the side chains of their pyranosyl substrates to approximate *gg* conformations and, we have argued, in doing so provide additional electrostatic stabilization to oxocarbenium ion-like transition states.^23,24^ Exceptions to this phenomenon mostly result from the enforcement of nonstandard conformations of the pyranoside ring on binding to the enzyme. Such nonstandard ring conformations either energetically disfavor the *gg* side-chain conformation or provide additional electrostatic stabilization from pseudoaxial C–O bonds, akin to the synthetic chemists’ concept of superarming,^22,25^ so minimizing the need for side-chain conformational control.^23,26^ Glycosidase inhibitors with appropriately restricted side-chain conformations, whether synthetic or natural, can show improved inhibitory properties over their unrestricted counterparts.^23,27,28^

**Figure 1:**
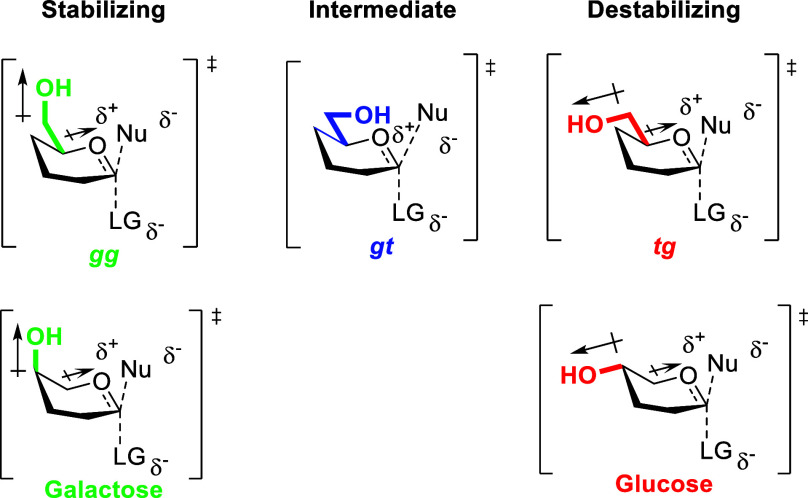
*Gauche,gauche* (*gg*), *gauche,trans* (*gt*), and *trans,gauche* (*tg*) conformations of sugar side chains illustrated for six-membered cyclic oxocarbenium ions and parallels of the gg and tg conformations with pseudoaxial and pseudoequatorial C–O bonds at the 4-position of galactosyl and glucosyl oxocarbenium ion-like transition states, respectively.

The concept of the control of substrate conformation by enzymes as a component of catalysis has a long history.^29–35^ It has also been strongly disputed by Warshel, who has argued that enzymes gain little by restraining the reactive fragments of their substrates^36^ and proposed that “the polar preorganization of enzyme active sites is the most important factor in enzyme catalysis” in large part by overcoming the reorganization energy of water molecules needed to electrostatically stabilize transition states.^37–39^

In this paper, we extend our PDB-driven analysis of side-chain conformational restriction by GHs and related enzymes to encompass GHs acting on furanosides, nucleoside hydrolases (NHs), nucleoside phosphorylases (NPs), nucleoside 2′-deoxyribosyltransferases (NDTs), 5′-methylthioadenosine/*S*-adenosylhomocysteine nucleosidases (MTANs), and 5′-methylthioadenosine phosphorylases (MTAPs) (Figure 2).

**Figure 2:**
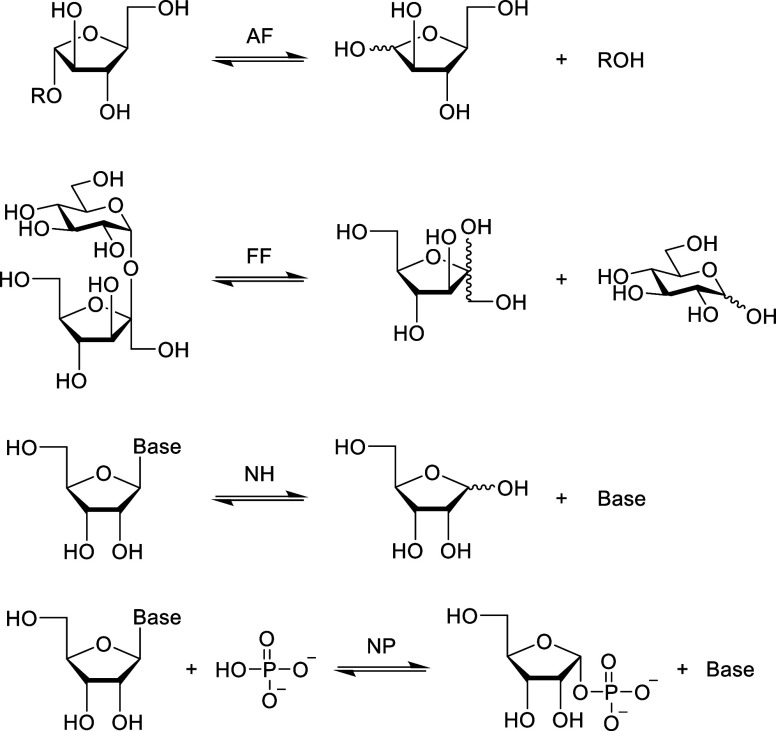
Transformations catalyzed by the l-arabinofuranosidases (AFs), d-fructofuranosidases (FFs), d-nucleosidases (NHs), and d-nucleoside phosphorylases (NPs).

We draw parallels with previous mechanistic work on the importance of the substrate side-chain hydroxy group in NHs and NPs, culminating in the development of the immucillins,^40,41^ to underline the importance of side-chain conformational control by GHs, GTs, and related enzymes. Finally, we argue that the restriction of side-chain conformation, and so substrate preorganization, by GHs, GTs, and related enzymes bridges the gap between classical physical organic-based substrate preorganization, with its emphasis on confining the reactive termini of the substrate and reducing the entropic penalty,^29–35^ and the electrostatic hypothesis propounded by Warshel and others in that the enzymes in question restrict the side-chain conformation to provide additional electrostatic stabilization to the transition state.^37–39,42^

## Results

The side-chain conformational equilibria of pentofuranosides have been investigated by Serianni and co-workers by classical NMR methods and by DFT calculations, leading to the approximate populations for the arabino and xylofuranosides set out in Figure 3.^43,44^ Serianni and co-workers also noted the coupling of ring and side-chain conformations in these systems,^44^ as was confirmed in a molecular dynamics (MD) study by Wang and Woods.^45^ In contrast, a subsequent MD study by Nester and Plazinski found little correlation between ring and side-chain conformations in the furanosides with ≤0.7 kcal mol^–1^ difference in the ribo and arabinofuranosides.^46^ Using a combination of VT-NMR and DFT methods, Lowary and co-workers determined the impact of side-chain conformation on anomeric reactivity in a series of 5-*O*-benzoyl-α- and β-d-lyxofuranosides and α- and β-furanosyl triflates finding a consistent *tg* > *gt* > *gg* stability order.^47^ The restriction of side-chain conformation in furanosides, whether by inclusion in a bicyclic system or simply by the introduction of an additional C–C bond, as in the hexofuranosides, has been demonstrated to impact anomeric reactivity.^48–52^ Overall, while the conformational equilibria and reactivity patterns do not follow exactly those found in the pyranosides, the same gross trends are found in the furanosides.

**Figure 3:**
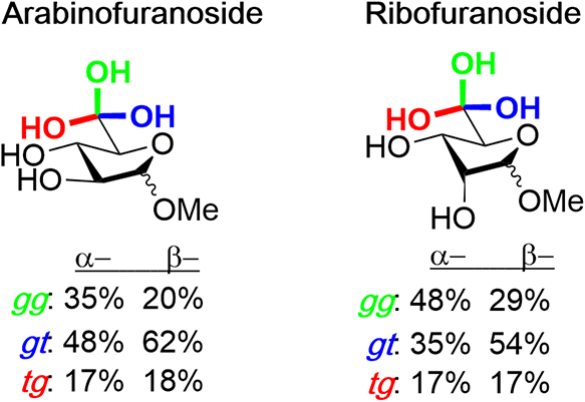
Side-chain conformation distributions of furanosides in solution.

Mining of the PDB using the Carbohydrate Active Enzymes database (CAZy, http://www.cazy.org)^53^ for structures of GHs binding furanosides and their analogs in their active (−1)^54^ site with resolution ≤2.5 Å, as described for the pyranosides,^23,24^ with data curation using the Privateer software^55^ to minimize errors,^56^ resulted in a data set (Tables 1 and 2) comprised of α- and β-l-arabinofuranosidases, β-d-fructofuranosidases, β-d-transfructofuranosidases, and one α-d-arabinofuranosidase/fructofuranosidase (see Supporting Information for full details). Clearly, the arabinofuranosidases (AFs), whether α- or β-, restrict the side chains of their substrates to the *gg* conformation, while the β-d-fructofuranosidases (FFs) enforce the *gt* conformation.

**Table 1:** Side-Chain and Ring Conformations of Furanoside Ligands Bound to l-Arabinose Processing Enzymes

enzyme function	*gg*	*gt*	*tg*	ring conformation	total	H-bonding to side chain
α-l-arabinofuranosidase	19	0	0	*E*_4_/*E*_2_	19	16
β-l-arabinofuranosidase	10	0	0	*E*_4_/^3^ *T*_2_	10	9

**Table 2:** Side-Chain and Ring Conformations of Furanoside Ligands Bound to d-Fructose Processing Enzymes

	side-chain conformation			anomeric side-chain conformation					
enzyme function	*gg*	*gt*	*tg*	*gg*	*gt*	*tg*	ring conformation	total	H-bonding to side chain
β-d-fructofuranosidase	0	28	0	1	26	1	*E*_3_	28	27
β-d-transfructofuranosidase	1	11	0	4	8	0	*E*_3_/^4^ *T*_3_	13^a^	7
α-d-arabinofuranosidase/α-d-fructofuranosidase	2	0	0	0	0	1	^3^ *T*_4_/*E*_5_ ^b^	2	2

a One structure has an ambiguous side-chain conformation.

b The arabinofuranosyl substrate has the ^3^ *T*_4_ ring conformation, while the fructofuranosyl substrate has the *E*_5_ ring conformation.

We next examined the NHs and NPs, whose mechanisms have been reviewed and also involve exploded oxocarbenium-ion-like transition states,^57–60^ restricting ourselves to the enzymes operating on nucleosides as substrates.^61^ By searching the Enzyme Commission (EC) numbers in the PDB and using the same criteria as described for the GHs above, we obtained the data set summarized in Table 3 for NHs (EC 3.2.2.1, EC 3.2.2.3, EC 3.2.2.8, and EC 3.2.2.22), NPs (EC 2.4.2.1, EC 2.4.2.2, EC 2.4.2.3, and EC 2.4.2.4), NDTs (EC 2.4.2.6), MTANs (EC 3.2.2.9 and 3.2.2.16), and MTAPs (EC 2.4.2.28).

**Table 3:** Side-Chain and Ring Conformations of Ligands in Complexes with d-Nucleoside Processing Enzymes

enzyme function	*gg*	*gt*	*tg*	ring conformation	total	H-bonding to side chain
d-nucleoside hydrolase (NH)	0	19	0	^4^ *E*	19	19
d-nucleoside phosphorylase (NP)	5	83	1	^4^ *E*	89	81
d-nucleoside 2′-deoxyribosyl-transferase (NDT)	0	6	0	*E*_O_	6	6
d-5′-methylthioadenosine/d-*S*-adenosylhomocysteine nucleosidase (MTAN)	1	3	41	^2^ *T*_3_/^4^ *E*	45	4
d-5′-methylthioadenosine phosphorylase (MTAP)	0	21	0	^4^ *E*/^O^ *E*	21	1

For the NHs, NPs, NDTs, and MTAPs, there is a very strong preference for binding the substrates with side-chain conformations that approximate the *gt* conformation, while for the MTANs, substrate binding with the *tg* conformation of the side chain is strongly preferred.

## Discussion

### Side-Chain Conformation

The contrast between the AFs (Table 1), which enforce the *gg* conformation of their side chains, and the FFs (Table 2), NHs, NPs, NDTs, and MTAPs (Table 3), which in contrast predominantly restrict the side chains of their substrates to the *gt* conformation, is striking. Yet a further contrast is apparent with the MTANs (Table 3), which have evolved to bind their substrates with the *tg* conformation of the side chains. We first address the AFs, FFs, NHs, NPs, and NDTs, all of which bear a hydroxy group in their substrate side chains, before returning to the MTANs and MTAPs with their sulfur-based substituents in the side chain of their substrates.

For the α-l-AFs when the bound ligand is l-arabinofuranose-derived or lacks an anomeric substituents, as in l-1,4-iminoarabinitol, it is very predominantly bound in the *E*_4_ conformation (Figure 4) with a single exception having the ^3^ *E* conformation. In contrast, when the anomeric substituent of the ligand is on the β-face, the *E*_2_ conformation is preferred, with the exception of a covalently bound adduct arising from invertive ring opening by an active-site glutamate residue of a carbasugar-derived α-cyclic sulfate that carries a residual sulfate on the α-face. All ligands bound in complex with an α-l-AF retain the *gg* conformation of the side chain, whether the anomeric configuration is α- or β-, and the implication is that the α-l-AFs bind their substrates as an *E*_4_ envelope with a pseudoequatorial side chain and restrict it to the *gg* conformation. After pseudorotation to the proximal ^3^ *E* envelope, still with a pseudoequatorial side chain restricted by hydrogen bonding to the *gg* conformation, invertive displacement at the anomeric center, whether by water or an active site residue, takes place through a ^3^ *T*_2_-like transition state ultimately providing a β-configured product in the *E*_2_ conformation. Finally, this initial product conformer may undergo pseudorotation to the closely related ^1^ *E* conformation, with the *gg* conformation of the side chain retained throughout the entire process (Figure 5). A single example of a difructose dianhydride I synthase/hydrolase exhibiting α-d-AF activity in complex with β-d-arabinofuranose (PDB 7V1W) has the ligand bound in an approximate ^3^ *T*_4_ conformation, midway between the *E*_4_ and ^3^ *E* envelopes, with its side chain restricted by hydrogen bonding to the *gg* conformation.^62^

**Figure 4:**
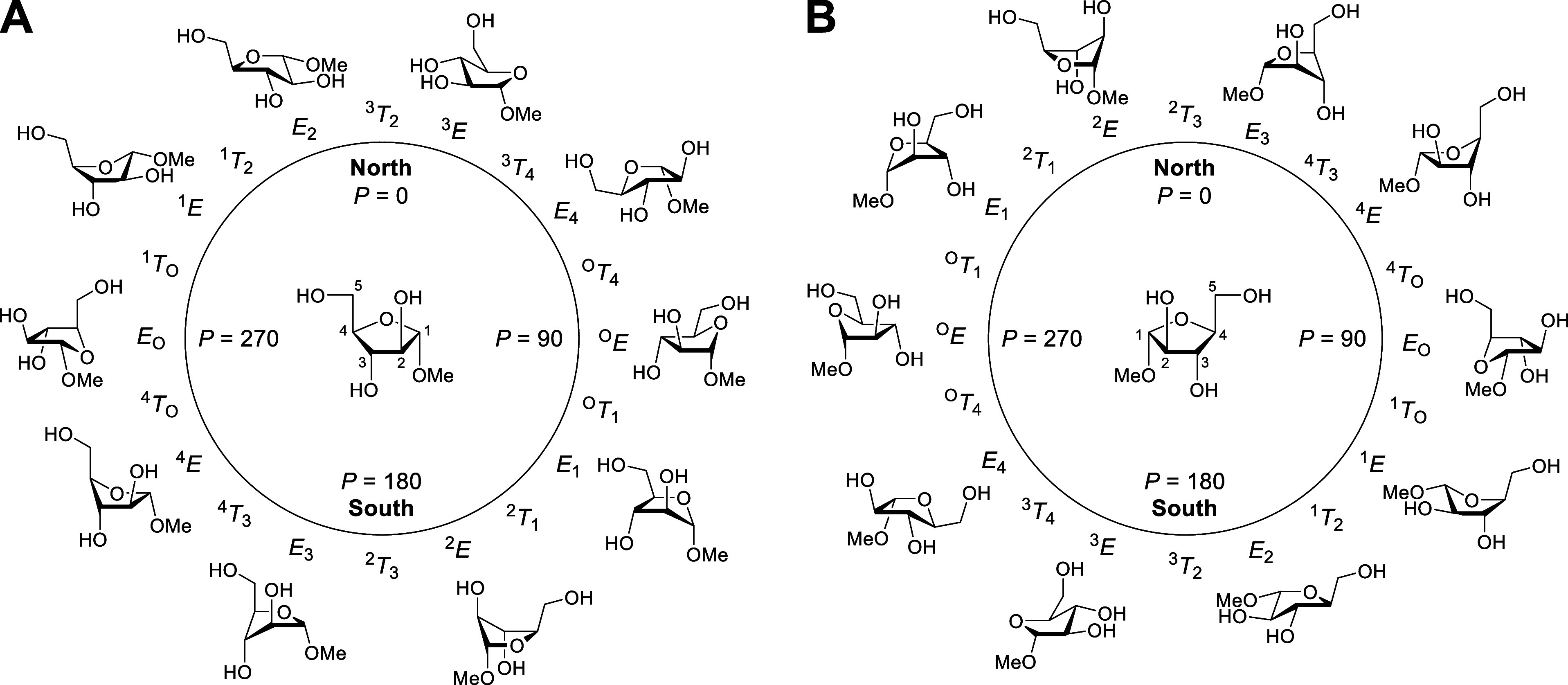
Pseudorotational wheels of α-d-arabinofuranoside (A) and α-l-arabinofuranoside (B) rings. Only the envelope conformations are shown. P, pseudorotational phase angle.

**Figure 5:**
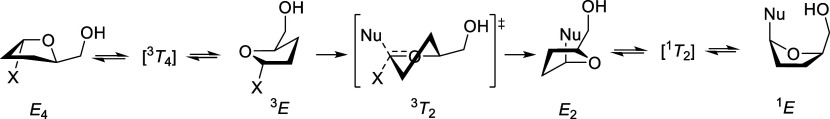
Apparent pseudorotational itinerary followed by substrates during hydrolysis by α-l-arabinofuranosidases. Hydroxy groups at the 2- and 3-positions are omitted for clarity.

For the β-l-AFs, arabinose-derived ligands with the β-anomeric configuration are bound mainly in either the ^3^ *E* or ^3^ *T*_2_ conformations, always with the side chain held in an approximate *gg* conformation by hydrogen bonding. On the other hand, β-l-AF structures with α-configured l-arabinofuranose derivatives, located in the form of covalent adducts with the protein, are found as either *E*_2_ or *E*_4_ envelopes, still with the side chain maintained in the *gg* conformation. This pattern holds even in recent examples that employ active-site cysteine residues as nucleophiles.^63,64^

Arabinofuranose and fructofuranose share the relative arabino-configuration of their nonanomeric positions (Figure 6), yet the β-d-FFs bind their fructose-derived ligands very predominantly in the *E*_3_ conformation (equivalent to the *E*_2_ conformation in the d-arabinofuranosides) and enforce the *gt* conformation of their side chains, both of which differ from the ligand conformation preferences of the AFs discussed above.

**Figure 6:**
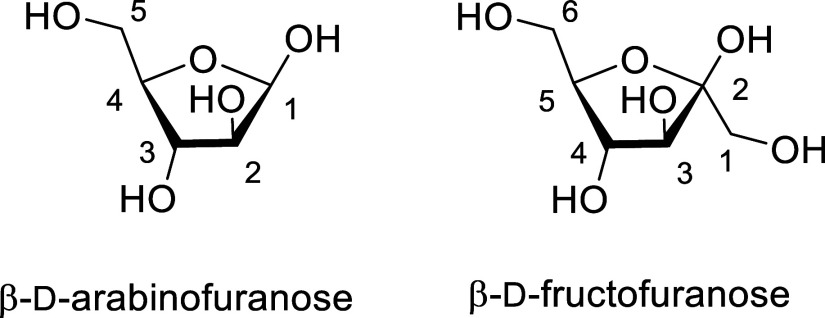
Structures of β-d-arabinofuranose and β-d-fructofuranose.

The difference in ring conformation between arabinofuranosides and fructofuranosides arises from the presence of the additional hydroxymethyl side chain at the anomeric center in the latter, hereinafter called the anomeric side chain, and the need to accommodate it in a pseudoequatorial position. Like the side chain itself, the anomeric side chain of the fructofuranosides can adopt three staggered conformations, which by analogy we dub the *gg*, *gt*, and *tg* conformations. Of the twenty-eight FF–ligand complexes located, all from GH32, twenty-seven adopt approximate *E*_3_ conformations, while the remaining one takes up the adjacent ^4^ *T*_3_ ring conformation. All of these twenty-eight structures enforce the *gt* conformation of the side chain and twenty-six of them similarly enforce approximate *gt* conformations on the anomeric side chain. There is therefore a strong preference among the β-d-FFs to bind either β-d-fructofuranose itself or β-d-fructofuranosides with an *E*_3_-like ring pucker with both side chains in the *gt* conformation (Figure 7). With the ring in the *E*_3_ envelope conformation, the anomeric substituent is pseudoaxial and would be subject to a steric clash with the 6-OH group were the side chain restricted to the *gg* conformation. We suggest that the preference for the *gt* conformation of the anomeric side chain arises because, in addition to providing modest stabilization to nascent positive charge at the transition state for hydrolysis, it benefits from stabilizing *gauche* interactions of the C1–O1 bond with both the C2–OR and C2–O5 bonds and has no steric *gauche* interactions with a C–C bond. In contrast, the *gg* and *tg* conformations both have only one stabilizing *gauche* interaction and suffer from one steric *gauche* interaction (Figure 7). It is apparent therefore that the FFs have evolved to reach a compromise holding both side chains of their substrates in the moderately activating *gt* conformation.

**Figure 7:**
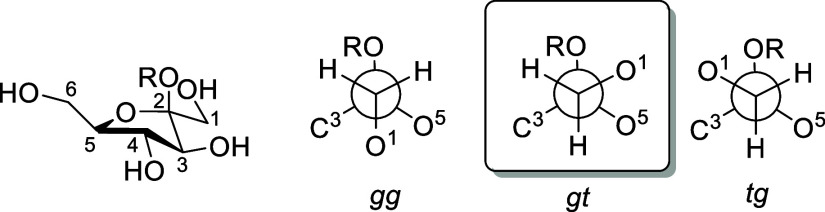
Predominant *E*_3_, *gt*, and *gt* conformations of β-d-fructofuranos­(id)­es in the active site of GH-32 β-d-fructofuranosidases and the staggered conformations about the C1–C2 bond.

Four *trans*-FFs from GH32 closely follow the FFs from the same family binding β-d-fructofuranose in either the *E*_3_ or the adjacent ^4^ *T*_3_ ring conformations with the *gt* conformation of the side chain and (with the exception of one *gg* example) the *gt* conformation of the anomeric side chain. Similarly, five *trans*-FFs from GH68 bind β-d-fructofuranose in the *E*_3_ conformation with the *gt* conformation of the side chain, and three of the five bind the anomeric side chain in the *gt* conformation, while the other two enforce the *gg* conformation of the anomeric side chain. Four structures meeting our selection criteria are available for the GH91 FF-transferases (PDB 9J4I, 5ZKU, 5ZLA, and 8HUI),^65–67^ which cleave difructofuranose from fructans initially providing various difructose anhydrides. The data from these structures, however, are ambiguous, perhaps because the imposed conformations reflect the need to position the nonreducing terminal residue of the substrates so as to act as a nucleophile in the formation of the anhydride products and are not analyzed further. Finally, a *trans*-FF from *Bifidobacterium dentium* belonging to the newly assigned GH class 172 additionally displays α-d-AF activity and α-d-FF activity. It binds β-d-fructofuranose in the *E*_5_ with the *gg* conformation of its side chain (PDB 7V1X) and β-d-arabinofuranose in the adjacent (taking account of the numbering shift) ^3^ *T*_4_ conformation with the *gg*-oriented side chain (PDB 7V1W) and so more closely resembles the AFs in other GH classes than the more common GH32 class of FFs and the GH91 *trans*-FFs. The anomeric side chain of the β-d-fructofuranose ligand in complex with the *B. dentium* *trans*-FF is hydrogen bonded in the deactivating *tg* conformation, thereby highlighting the difference with the FFs from GH32 and the *trans*-FFs from GH classes 68 and 91.

The NHs and the NPs from both the NP-I and NP-II families, with their distinctly different folds,^68^ almost exclusively bind their ribo-configured substrates and substrate analogs with the *gt* conformation of the side chain and show a very significant preference for the ^4^ *E* ring conformation, with occasional exceptions having the *E*_3_, *E*_O_, or ^2^ *T*_3_ ring conformations. All ring conformations are therefore in the southwest quadrant of the pseudorotational wheel for d-furanosides and importantly place the side chain in a pseudoaxial orientation (Figure 8). The situation for the NHs and NPs therefore closely parallels that of the FFs, with the furanoside ring bound in a conformation that excludes the *gg* side-chain conformation because of the steric clash it would engender with the leaving group at the anomeric position, leading to the imposition of the second most activating *gt* side-chain conformation. Finally, although the number of structures is limited (Table 3), NDTs from both classes I and II, which catalyze the transfer of the 2′-deoxyribosyl moiety from a nucleoside to an acceptor nucleobase,^69,70^ bind substrate analogs and the nucleoside products with a preference for the *E*_O_ ring conformation and the *gt* side-chain conformation.^71–73^

**Figure 8:**
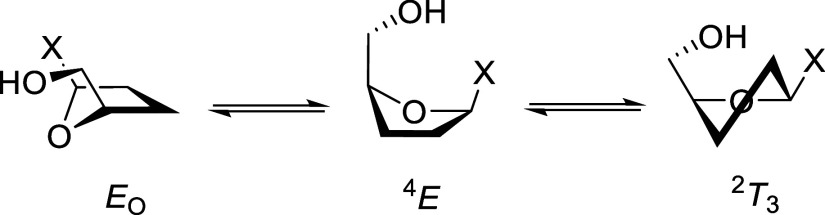
Predominant bound ring and side-chain conformations of the ribonucleoside hydrolase and ribonucleoside phosphorylase substrates.

Turning to the MTANs and MTAPs in which the C5′–OH bond of the substrates is replaced by a longer and less electron-withdrawing C5′–SR bond, the data reveal that MTAN substrates and their analogs are mostly bound in the ^2^ *T*_3_ and ^4^ *E* ring conformations like the NHs and NPs, but with their side chains almost exclusively restricted to the *tg* conformation (Figure 9). The MTAPs on the other hand bind their substrates and substrate analogs with predominantly the ^4^ *E* and ^O^ *E* ring conformations and the *gt* side chain conformation. The more diffuse electron density around the 5′-sulfur atom of the MTAN and MTAP substrates and their longer 5′-C–S bonds combine to reduce any electrostatic transition-state stabilization afforded by side chains in the *gg* or *gt* conformations. At the same time, the lower electronegativity of sulfur compared to oxygen renders the *tg* side chain conformation less destabilizing toward nascent positive charge at the transition state, such that overall there is little to be gained for the MTANs and MTAPs by restricting the side chains of their substrates to the *gg* or *gt* conformations. Presumably, the MTANs have evolved to bind their substrates in the *tg* conformation to remove the steric bulk of the 5′-residue from the vicinity of the reaction center particularly when functioning as *S*-adenosylhomocysteine nucleosidases, while the MTAPs have retained the preference for the *gt* conformation enjoyed by the NPs in general.

**Figure 9:**
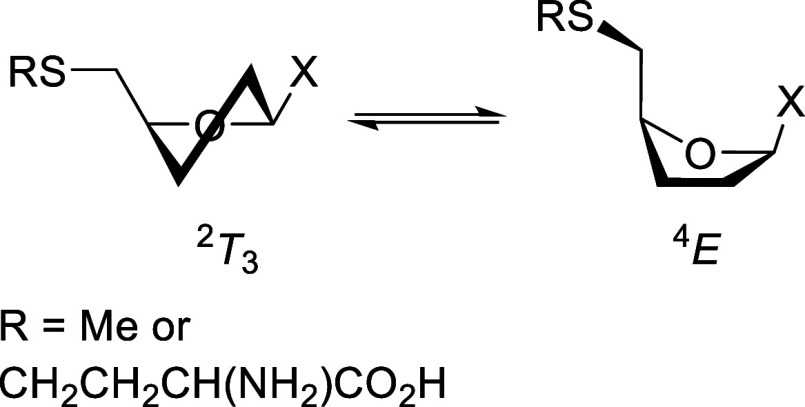
Predominant bound ring and *tg* side-chain conformations of the 5′-methylthioadenosine/*S*-adenosylhomocysteine nucleosidase substrates.

Overall, examination of the crystallographic record provides strong evidence that AFs, FFs, NHs, NPs, and NDTs restrict the conformation of their substrates by hydrogen bonding to provide electrostatic stabilization by the 5′-hydroxy group of their substrates to nascent positive charge at the anomeric center and in doing so enhance catalysis. When the substrate is bound in a ring conformation that readily accommodates it as in the AFs, the more stabilizing *gg* conformation of the side chain is preferred, as is found in the vast majority of the α- and β-glucopyranosidases and, with certain well-defined exceptions, the α- and β-mannopyranosidases. When the substrate is bound in a ring conformation in which the energy of the *gg* side-chain conformation would be increased by a steric clash with the leaving group (FFs, NHs, and NPs), the side chain is instead preferentially bound in the second most activating *gt* conformation. This compromise is akin to that found in the α-galactopyranosidases where the *gg* side-chain conformation of the substrate is higher in energy due to dipolar repulsion with the C4–O4 bond, resulting in the evolution of the preference for binding in the second most activating *gt* conformation.^23^ We consider the MTANs, with their preference for binding their substrates with the *tg* side-chain conformation because of the relative absence of electrostatic stabilization available from the side-chain substituent, as exceptions that reinforce the overall rule.

### Correlation with the Literature on the Role of the Substrate Side Chain in the Action of NHs and NPs

Our initial search of the PDB leading to the recognition that pyranosidases restrict the side-chain conformation of their substrates to enhance catalysis^23^ was driven by our insight into the role of the side chain in chemical glycosylation reactions. The results presented above suggest that nature widely applies the same phenomenon to furanosidases, nucleosidases, and nucleoside phosphorylases. The case for side-chain conformational control by NHs and NPs is strongly supported by a considerable body of biochemical and physical organic studies on individual enzymes over many years. Thus, Schramm and co-workers, studying the trypanosome *Crithidia fasciculata* NH, found (i) that 5′-deoxyinosine is not a substrate and (ii) a secondary ^3^H kinetic isotope effect at the 5′-position of the substrate inosine leading them to implicate a “transition-state function for the 5′-hydroxyl” and to initially propose hyperconjugative stabilization of the developing charge at C4′ by a C5′–H5′ bond from a *gt* conformation of the side chain.^74–76^ In a subsequent computational analysis, this transition-state hypothesis was revised to place the side chain in an approximate *gg* conformation in such a manner that “more electrostatic binding energy could be developed by placing a negatively charged group or suitable dipole over the ribose ring”, leading the authors to state that “structural features of the transition state, sometimes remote from the site of bond breaking and forming, are likely to be used to distribute or localize charge development to optimize structures which can be stabilized at enzymatic transition states”.^77,78^ A subsequent X-ray structure of this NH in complex with the inhibitor *p*-aminophenyliminoribitol generally confirmed this hypothesis and revealed a O5′–C5′–C4′–N4′ torsion angle of 336°, i.e., midway between the *gt* conformation and one in which O4′ and O5′ are eclipsed with stabilization of the positive charge by a “neighboring group” (Figure 10A; PDB 2MAS).^79^ Steyaert and co-workers studied the *Trypanosoma vivax* NH and similarly found the 5′-deoxy analog of the natural substrate adenosine to be a very poor substrate and calculated that the 5′-hydroxy group contributes 5.4 kcal mol^–1^ to the catalytic efficiency of hydrolysis of adenosine. Working with a D10A mutant, lacking a catalytic aspartate residue, they were able to determine structures of the complex with the natural substrate inosine, representing a snapshot of pretransition-state species, and of that with the inhibitor 3-deazainosine: in both structures (PDB 1KIC and 1KIE), the side chain was held in an approximate *gt* conformation.^80^ The complex of the natural *T. vivax* NH with the transition-state analog inhibitor immucillin H also retains the approximate *gt* conformation of the inhibitor side chain, although with some deformation toward eclipsing with the C4′–O4′ bond (Figure 10B; PDB 2FF2),^81^ albeit alternative rationalizations were proposed for the role of the 5′-hydroxy group on the basis of computational work.^82^ The importance of the substrate 5′-hydroxy group to hydrolysis has also been demonstrated for the *Trypanosoma brucei* NH, although in this case, its contribution was considered to arise more from formation of the Michaelis complex than to catalysis.^83^ In contrast to the NHs, with their significant secondary ^3^H KIEs and requirements for the presence of the 5′-hydroxy group, the MTANs show only small secondary KIEs at the 5′-position, attributed to hyperconjugation with sulfur,^84–86^ and are able to process their 5′-unsubstituted congeners.^87^

**Figure 10:**
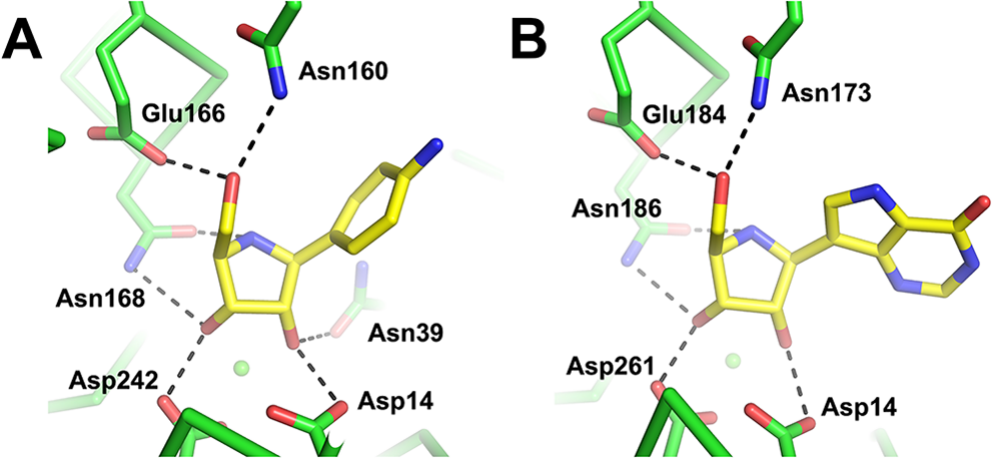
Active site–ligand interactions from (A) PDB 2MAS and (B) PDB 2FF2 showing protein–ligand H-bonding holding the ligand side chain in the *gt* conformation.

The human purine NP (PNP) binds inosine derivatives carrying modifications at the 5′-position, such as alkylthio, halo, and deoxy derivatives, but they are very poor substrates.^88,89^ Secondary ^3^H KIE studies on the hydrolysis of inosine by human PNP in the absence of phosphate revealed the involvement of the 5′-position in the transition state, prompting the authors to propose an analogous transition state to that for the NHs with the side chain in an approximate *gt* conformation.^90^ These KIEs, together with a series of crystallographic snapshots of PNPs with the inhibitor immucillin H and inorganic phosphate (Figure 11A; PDB 1B8O) and the product α-d-ribofuranosyl phosphate (Figure 11B; PDB 1A9T), led to the proposition of an early oxocarbenium ion-like transition state. In this transition state, the side chain takes up a conformation midway between *gt* and *gg* in which the C5′–O5′ bond eclipses the C4′–O4′ bond so as, in combination with the electron density on the α-face provided by the phosphate nucleophile, to “favor the release of electrons from O4′, cleavage of the C1′–N9 bond, and formation of the ribooxocarbenium ion transition state (electron push)”.^91^ Multiple subsequent structures of PNPs with substrates or inhibitors support this analysis, ultimately leading to an understanding of the mechanism in which the 5′-hydroxy group is hydrogen bonded to His257 at the level of the Michaelis complex with moderate distortion toward the transition state. Finally, at the transition state, the 5′-hydroxy group approaches an eclipsed conformation with O4′ and provides “neighboring group participation” at the transition state.^92^ Transition path sampling simulations were used to probe the approach of O5′ to O4′ and showed it to reach a minimum of approximately 2.85 Å at the transition state. The extensive studies of Schramm and co-workers toward the characterization of the PNP mechanism have been reviewed.^41,93^

**Figure 11:**
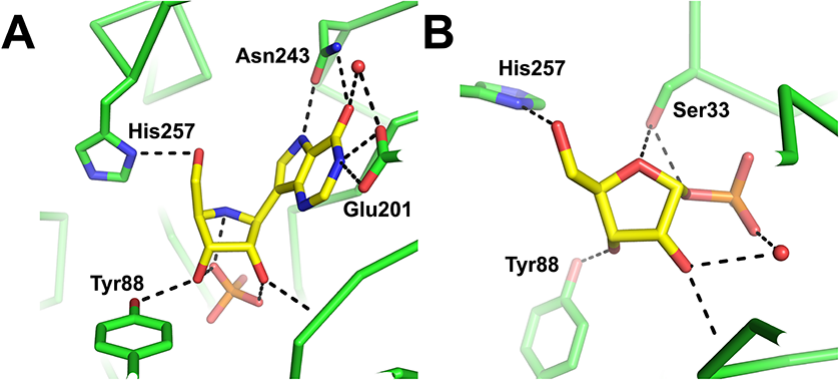
Active site–ligand interactions from (A) PDB 1B8O and (B) PDB 1A9T.

Schramm’s concept of a push from the electron density on O5′ when eclipsing the C4′–O4′ bond that facilitates departure of the base from C1′ in the NHs and NPs and our concept of side-chain preorganization into the arming *gg* conformation, and when that is sterically inaccessible the *gt* conformation, are evidently two faces of the same coin. Any subtle differences between the two descriptions are attributable to conformations imposed by the enzymes on the ring and possibly to the extent of charge delocalization onto the ring oxygen at the transition state. Setting aside these minor variations on the theme, it is difficult to escape the conclusion that the majority of GHs, NHs, and NPs and, as is clear from our database searches,^24^ transglycosidases and Leloir GTs have evolved to bind their substrates so that their side chains are conformationally preorganized to provide additional electrostatic stabilization to oxocarbenium ion-like transition states.

### Provision of Electrostatic Stabilization to Transition States by Substrate Preorganization

We now argue that side-chain preorganization by this broad group of glycoside and nucleoside processing enzymes bridges the gap between Warshel, who has strongly disputed the concept that enzymes employ the conformation restriction of their substrates to enhance catalysis,^36^ and multiple authors who have strongly advocated for such restriction often on the basis of physical organic studies of model systems.^29–35^ We argue that the restriction of substrate side-chain conformation by GHs and related enzymes to provide electrostatic stabilization to at least partially positively charged transition states is consistent with Warshel’s concept that, in addition to providing correctly positioned catalytic residues, enzymes achieve catalysis largely by preorganizing the dipoles of active-site polar groups to maximize electrostatic stabilization of transition states.^38,39,42^ Thus, adopting Warshel’s analysis,^38^ in the uncatalyzed reaction in aqueous solution as the substrate passes from the reactant state to the transition state, it suffers a change in charge distribution, which causes the dipoles of the surrounding water molecules to orient themselves so as to stabilize the charges on the transition state, for which there is an energy penalty, known as the reorganization energy. In the enzymic reaction, on the other hand, active-site polar residues are already correctly aligned and do not have to undergo significant reorganization to stabilize charges in the transition state. As the degree of stabilization of the charge on the transition state by reorganized water and active-site residues is comparable, a significant contribution to catalysis is the removal of reorganization energy for the reaction in water. It is apparent that active-site residues in GHs and related enzymes are preorganized to restrict the substrate side chain to the *gg* conformation where it is poised to provide electrostatic stabilization to the transition state. When the *gg* conformation is sterically disfavored because of the conformation imposed on the ring at the transition state, the enzyme is preorganized to select the second most stabilizing *gt* conformation. Roughly speaking, the penalty paid for restricting the side-chain conformation will be comparable to the reorganization energy of a single appropriately placed water molecule in the aqueous reaction or to the retention and conformational restriction of a single water molecule in the active-site in place of the side-chain hydroxy group. Of note in this regard is the minimal effect that removal of the hydroxymethyl side chain from the substrate has on the hydrolysis of β-d-galactopyranosyl substrates by the *Escherichia coli* (*lacZ*) β-galactosidase,^94^ which was later rationalized in terms of possible binding of a water molecule in the place of the hydroxymethyl side chain.^95^

Regarding the magnitude of the transition-state stabilization afforded by the conformationally restricted side-chain hydroxy group, Steyaert and co-workers estimate that relative to the 5′-deoxy analog, the 5′-hydroxy group contributes 5.4 kcal mol^–1^ to the catalytic efficiency of adenosine hydrolysis by the *T. vivax* NH,^80^ which likely defines an upper limit. This is because deoxygenation or deoxyhalogenation of the side chain leaves a methyl or halomethyl residue that is likely too large to allow a water molecule to fill the cavity. Consequently, the reduction in activity due to the loss of electrostatic transition-state stabilization on deletion of the side-chain hydroxy group will be accentuated by the reduction in affinity arising from the absence of hydrogen bonding to the active-site residue(s) responsible for the restriction of the side-chain conformation in the native substrate. The beneficial effect of the substrate side-chain hydroxy group in various pyranosidases that are similarly found to process 6-deoxy analogs more slowly than the actual substrates^96–104^ has been attributed to hydrogen bonding with the active-site, but as crystallographic studies have shown hydrogen bonding with restriction of conformation of the side chain typically begins at the level of the enzyme substrate complex and is retained through the transition state to the enzyme product complex.^23^ This suggests that the hydrogen bonding to the side chain per se contributes mainly to *K*_M_. Contributions to *k*_cat_ on the other hand will arise from the electrostatic interaction of the side-chain hydroxy group with the partially positively charged transition state and any minor conformational adjustments of the side-chain conformation required to optimize it, as found by Schramm in his studies on mechanism of nucleoside phosphorylation by human PNP.^92^

Finally, we note that the concept of substrate preorganization to provide electrostatic stabilization to the transition state is not restricted to the substrate side chain. Thus, the GH47 α-mannopyranosidases benefit from chelation of Ca^2+^ to bind their substrates in a ^3^ *S*_1_ twist boat conformation on the way to the proximal ^3^ *H*_4_ half chair transition state for hydrolysis in which O3 and O4 are pseudoaxial and provide maximal electrostatic stabilization.^26^ Yet other glycosidases appear to use steric hindrance to force their substrates into conformations enriched in pseudoaxial hydroxy groups poised to provide electrostatic transition-state stabilization.^26^

## Conclusion

Mining of the PDB reveals that the arabinofuranosidases enforce the *gg* conformation on the side chains of their substrates, while the fructofuranosidases, ribonucleoside hydrolases, phosphorylases, and transferases, for which the *gg* conformation is sterically hindered, very largely restrict the side-chain conformations of their substrates to the *gt* conformation. Hydrolases that catalyze reactions via partially positively charged furanosyl oxocarbenium ion-like transition states therefore follow the pattern described earlier for glycoside hydrolases, transglycosidases, and Leloir glycosyltransferases acting on pyranosyl substrates by restricting side-chain conformation to provide additional electrostatic stabilization to the transition state. These conclusions are supported by extensive literature studies on the importance of the substrate 5′-hydroxyl group in C–N bond cleavage reactions by nucleoside hydrolases and phosphorylases and are consistent with the electrostatic basis for enzyme catalysis propounded by Warshel and others.^37,38,42^

## Supplementary Material



## Notes

Additional information on Privateer analysis and tables of raw crystal structure data (PDF)

## References

[1] Wolfenden R. (2006). Degrees of
Difficulty of Water-Consuming Reactions in the Absence of Enzymes. Chem. Rev..

[2] Jongkees S. A. K., Withers S. G. (2014). Unusual Enzymatic
Glycoside Cleavage Mechanisms. Acc. Chem. Res..

[3] Collén P. N., Jeudy A., Sassi J.-F., Groisillier A., Czjzek M., Coutinho P. M., Helbert W. (2014). A Novel Unsaturated
β-Glucuronyl Hydrolase Involved in Ulvan Degradation Unveils
the Versatility of Stereochemistry Requirements in Family GH105. J. Biol. Chem..

[4] Jongkees S. A. K., Yoo H., Withers S. G. (2014). Mechanistic Investigations of Unsaturated
Glucuronyl Hydrolase from *Clostridium perfringens*. J. Biol. Chem..

[5] Zechel D. L., Withers S. G. (2000). Glycosidase Mechanisms: Anatomy of a Finely Tuned Catalyst. Acc. Chem. Res..

[6] Davies G. J., Planas A., Rovira C. (2012). Conformational
Analyses of the Reaction
Coordinate of Glycosidases. Acc. Chem. Res..

[7] Lo J. M., Kung C.-C., Cheng T.-J. R., Wong C.-H., Ma C. (2025). Structure-Based
Mechanism and Specificity of Human Galactosyltransferase β3GalT5. J. Am. Chem. Soc..

[8] Ardèvol A., Iglesias-Fernández J., Rojas-Cervellera V., Rovira C. (2016). The Reaction Mechanism of Retaining Glycosyltransferases. Biochem. Soc. Trans..

[9] Colombo C., Bennet A. J. (2019). The Physical Organic
Chemistry of Glycopyranosyl Transfer
Reactions in Solution and Enzyme-Catalyzed. Curr. Opin. Chem. Biol..

[10] Crich D. (2010). Mechanism
of a Chemical Glycosylation. Acc. Chem. Res..

[11] Adero P. O., Amarasekara H., Wen P., Bohé L., Crich D. (2018). The Experimental Evidence in Support of Glycosylation Mechanisms
at the S_N_1-S_N_2 Interface. Chem. Rev..

[12] Andreana P. R., Crich D. (2021). Guidelines for *O*-Glycoside Formation from First
Principles. ACS Cent. Sci..

[13] Fraser-Reid B., Wu Z. C., Andrews W., Skowronski E., Bowen J. P. (1991). Torsional Effects in Glycoside Reactivity: Saccharide
Couplings Mediated by Acetal Protecting Groups. J. Am. Chem. Soc..

[14] Crich D., Sun S. (1996). Formation of β-Mannopyranosides
of Primary Alcohols Using the
Sulfoxide Method. J. Org. Chem..

[15] Crich D., Sun S. (1997). Direct Synthesis of β-Mannopyranosides by the Sulfoxide Method. J. Org. Chem..

[16] Jensen H.
H., Nordstrøm L. U., Bols M. (2004). The Disarming Effect of the 4,6-Acetal
Group on Glycoside Reactivity: Torsional or Electronic. J. Am. Chem. Soc..

[17] Moumé-Pymbock M., Furukawa T., Mondal S., Crich D. (2013). Probing the Influence
of a 4,6-*O*-Acetal on the Reactivity of Galactopyranosyl
Donors: Verification of the Disarming Influence of the *Trans-Gauche* Conformation of C5-C6 Bonds. J. Am. Chem.
Soc..

[18] Woods R. J., Andrews C. W., Bowen J. P. (1992). Molecular
Mechanical Investigations
of the Properties of Oxacarbenium Ions. Application to Glycoside Hydrolysis. J. Am. Chem. Soc..

[19] Miljkovic M., Yeagley D., Deslongchamps P., Dory Y. L. (1997). Experimental and
Theoretical Evidence of Through-Space Electrostatic Stabilization
of the Incipient Oxocarbenium Ion by an Axially Oriented Electronegative
Substituent During Glycopyranoside Acetolysis. J. Org. Chem..

[20] Bülow A., Meyer T., Olszewski T. K., Bols M. (2004). The C-4 Configuration
as a Probe for the Study of Glycosidation Reactions. Eur. J. Org. Chem..

[21] Smith D. M., Woerpel K. A. (2006). Electrostatic Interactions
in Cations and Their Importance
in Biology and Chemistry. Org. Biomol. Chem..

[22] Pedersen C. M., Marinescu L. G., Bols M. (2011). Glycosyl Donors in Unusual Conformations
- Influence on Selectivity and Reactivity. C.
R. Chim..

[23] Quirke J. C. K., Crich D. (2020). Glycoside Hydrolases
Restrict the Side Chain Conformation
of Their Substrates to Gain Additional Transition State Stabilization. J. Am. Chem. Soc..

[24] Quirke J. C. K., Crich D. (2021). Side Chain Conformation Restriction in the Catalysis
of Glycosidic Bond Formation by Leloir Glycosyltransferases, Glycoside
Phosphorylases, and Transglycosidases. ACS Catal..

[25] McDonnell C., López O., Murphy P. V., Fernández
Bolaños J. G., Hazell R. G., Bols M. (2004). Conformational Effects
on Glycoside
Reactivity: Study of the High Reactive Conformer of Glucose. J. Am. Chem. Soc..

[26] Quirke J.
C. K., Crich D. (2021). GH47 and Other
Glycoside Hydrolases Catalyze Glycosidic
Bond Cleavage with the Assistance of Substrate Super-Arming at the
Transition State. ACS Catal..

[27] Tseng P.-S., Ande C., Moremen K. W., Crich D. (2023). Influence
of Side Chain
Conformation on the Activity of Glycosidase Inhibitors. Angew. Chem., Int. Ed..

[28] Gloster T. M., Madsen R., Davies G. J. (2006). Dissection of Conformationally Restricted
Inhibitors Binding to a β-Glucosidase. ChemBioChem.

[29] Page M. I., Jencks W. P. (1971). Entropic Contributions
to Rate Accelerations in Enzymic
and Intramolecular Reactions and the Chelate Effect. Proc. Natl. Acad. Sci. U.S.A..

[30] Jencks W. P. (1975). Binding
Energy, Specificity, and Enzymic Catalysis: The Circe Effect. Adv. Enzymol. Relat. Areas Mol. Biol..

[31] Menger F. M. (1985). On the
Source of Intramolecular and Enzymic Reactivity. Acc. Chem. Res..

[32] Menger F. M. (1993). Enzyme
Reactivity from an Organic Perspective. Acc.
Chem. Res..

[33] Menger F. M. (2005). An Alternative
View of Enzyme Catalysis. Pure Appl. Chem..

[34] Benkovic S. J., Hammes G. G., Hammes-Schiffer S. (2008). Free-Energy
Landscape of Enzyme Catalysis. Biochemistry.

[35] Bruice T. C., Lightstone F. C. (1999). Ground State and Transition State Contributions to
the Rates of Intramolecular and Enzymatic Reactions. Acc. Chem. Res..

[36] Shurki A., Strajbl M., Villà J., Warshel A. (2002). How Much Do Enzymes
Really Gain by Restraining Their Reacting Fragments?. J. Am. Chem. Soc..

[37] Warshel A. (1998). Electrostatic
Origin of the Catalytic Power of Enzymes and the Role of Preorganized
Active Sites. J. Biol. Chem..

[38] Warshel A., Sharma P. K., Kato M., Xiang Y., Liu H., Olsson M. H. M. (2006). Electrostatic Basis for Enzyme Catalysis. Chem. Rev..

[39] Jindal G., Warshel A. (2017). Misunderstanding the
Preorganization Concept Can Lead
to Confusions About the Origin of Catalysis. Proteins.

[40] Schramm V. L. (2015). Transition
States and Transition State Analogue Interactions with Enzymes. Acc. Chem. Res..

[41] Evans G. B., Tyler P. C., Schramm V. L. (2018). Immucillins in Infectious Diseases. ACS Infect. Dis..

[42] Ruiz-Perniá J.
J., Świderek K., Bertran J., Moliner V., Tuñón I. (2024). Electrostatics
as a Guiding Principle in Understanding and Designing Enzymes. J. Chem. Theory Comput..

[43] Wu G. D., Serianni A. S., Barker R. (1983). Stereoselective Deuterium Exchange
of Methylene Protons in Methyl Tetrofuranosides: Hydroxymethyl Group
Conformations in Methyl Pentofuranosides. J.
Org. Chem..

[44] Cloran F., Carmichael I., Serianni A. S. (2001). 2-Deoxy-β-D-Erythro-Pentofuranose:
Hydroxymethyl Group Conformation and Substituent Effects on Molecular
Structure, Ring Geometry, and NMR Spin-Spin Coupling Constants from
Quantum Chemical Calculations. J. Am. Chem.
Soc..

[45] Wang X., Woods R. J. (2016). Insights into Furanose
Solution Conformations: Beyond
the Two-State Model. J. Biomol. NMR.

[46] Nester K., Plazinski W. (2020). Deciphering the Conformational Preferences
of Furanosides.
A Molecular Dynamics Study. J. Biomol. Struct.
Dyn..

[47] Callam C. S., Gadikota R. R., Krein D. M., Lowary T. L. (2003). 2,3-Anhydrosugars
in Glycoside Bond Synthesis. NMR and Computational Investigations
into the Mechanism of Glycosylations with 2,3-Anhydrofuranosyl Glycosyl
Sulfoxides. J. Am. Chem. Soc..

[48] Zhu X., Kawatkar S. P., Rao Y., Boons G.-J. (2006). Practical Approach
for the Stereoselective Introduction of β-Arabinofuranosides. J. Am. Chem. Soc..

[49] Ishiwata A., Akao H., Ito Y. (2006). Stereoselective
Synthesis of a Fragment
of Mycobacterial Arabinan. Org. Lett..

[50] Crich D., Pedersen C. M., Bowers A. A., Wink D. J. (2007). On the Use of 3,5-Di-*O*-Benzylidene and 3,5-Di-*O*-(Di-*tert*-Butylsilylene)-2-*O*-Benzylarabinothiofuranosides
and Their Sulfoxides as Glycosyl Donors for the Synthesis of β-Arabinofuranosides:
Importance of the Activation Method. J. Org.
Chem..

[51] Knapp S., Thakur V. V., Madduru M. R., Malolanarasimhan K., Morriello G. J., Doss G. A. (2006). Short Synthesis
of Octosyl Nucleosides. Org. Lett..

[52] Siyabalapitiya
Arachchige S., Crich D. (2022). Side Chain Conformation and Its Influence
on Glycosylation Selectivity in Hexo- and Higher Carbon Furanosides. J. Org. Chem..

[53] Lombard V., Golaconda R. H., Drula E., Coutinho P. M., Henrissat B. (2013). The Carbohydrate-Active
Enzymes Database (CAZy) in 2013. Nucleic Acid
Res..

[54] Davies G. J., Wilson K. S., Henrissat B. (1997). Nomenclature
for Sugar-Binding Subsites
in Glycosyl Hydrolases. Biochem. J..

[55] Agirre J., Iglesias-Fernández J., Rovira C., Davies G. J., Wilson K. S., Cowtan K. (2015). Privateer: Software for the Conformational
Validation of Carbohydrate Structures. Nat.
Struct. Biol..

[56] Agirre J., Davies G. J., Wilson K. S., Cowtan K. (2015). Carbohydrate Anomalies
in the PDB. Nat. Chem. Biol..

[57] We exclude from consideration enzymes processing furanosyl C–N bonds in nucleotides because of the presence of the phosphate group at the 5′-position.

[58] Berti P. J., McCann J. A. B. (2006). Toward a Detailed Understanding of Base Excision Repair
Enzymes: Transition State and Mechanistic Analyses of *N*-Glycoside Hydrolysis and *N*-Glycoside Transfer. Chem. Rev..

[59] Schramm V. L. (2018). Enzymatic
Transition States and Drug Design. Chem. Rev..

[60] Bzowska A., Kulikowska E., Shugar D. (2000). Purine Nucleoside Phosphorylases:
Properties, Functions, and Clinical Aspects. Pharmacol. Ther..

[61] We exclude nucleotides from our analysis because of the phosphorylated nature of their side chain hydroxyl groups.

[62] Kashima T., Okumura K., Ishiwata A., Kaieda M., Terada T., Arakawa T., Yamada C., Shimizu K., Tanaka K., Kitaoka M., Ito Y., Fujita K., Fushinobu S. (2021). Identification
of Difructose Dianhydride I Synthase/Hydrolase from an Oral Bacterium
Establishes a Novel Glycoside Hydrolase Family. J. Biol. Chem..

[63] McGregor N. G. S., Coines J., Borlandelli V., Amaki S., Artola M., Nin-Hill A., Linzel D., Yamada C., Arakawa T., Ishiwata A., Ito Y., van der Marel G. A., Codée J. D. C., Fushinobu S., Overkleeft H. S., Rovira C., Davies G. J. (2021). Cysteine Nucleophiles
in Glycosidase
Catalysis: Application of a Covalent β-L-Arabinofuranosidase
Inhibitor. Angew. Chem., Int. Ed..

[64] Borlandelli V., Offen W., Moroz O., Nin-Hill A., McGregor N., Binkhorst L., Ishiwata A., Armstrong Z., Artola M., Rovira C., Davies G. J., Overkleeft H. S. (2023). β-L-Arabinofurano-Cyclitol
Aziridines Are Covalent Broad-Spectrum Inhibitors and Activity-Based
Probes for Retaining β-L-Arabinofuranosidases. ACS Chem. Biol..

[65] Chen G., Wang Z.-X., Yang Y., Li Y., Zhang T., Ouyang S., Zhang L., Chen Y., Ruan X., Miao M. (2024). Elucidation of the Mechanism Underlying the Sequential Catalysis
of Inulin by Fructotransferase. Int. J. Biol.
Macromol..

[66] Yu S., Shen H., Cheng Y., Zhu Y., Li X., Mu W. (2018). Structural And Functional Basis of
Difructose Anhydride III Hydrolase,
Which Sequentially Converts Inulin Using the Same Catalytic Residue. ACS Catal..

[67] Cheng M., Hou X., Huang Z., Chen Z., Ni D., Zhang W., Rao Y., Mu W. (2024). Structural Insights into the Catalytic Cycle of Inulin
Fructotransferase: From Substrate Anchoring to Product Release. J. Agric. Food Chem..

[68] Pugmire M. J., Ealick S. E. (2002). Structural Analyses Reveal Two Distinct Families of
Nucleoside Phosphorylase. Biochem. J..

[69] Short S. A., Armstrong S. R., Ealick S. E., Porter D. J. T. (1996). Active Site Amino
Acids That Participate in the Catalytic Mechanism of Nucleoside 2-Deoxyribosyltransferase. J. Biol. Chem..

[70] Armstrong S. R., Cook W. J., Short S. A., Ealick S. E. (1996). Crystal Structures
of Nucleoside 2-Deoxyribosyltransferase in Native and Ligand-Bound
Forms Reveal Architecture of the Active Site. Structure.

[71] Anand R., Kaminski P. A., Ealick S. E. (2004). Structures
of Purine 2′-Deoxyribosyltransferase,
Substrate Complexes, and the Ribosylated Enzyme Intermediate at 2.0
Å Resolution. Biochemistry.

[72] Salihovic A., Ascham A., Taladriz-Sender A., Bryson S., Withers J. M., McKean I. J. W., Hoskisson P. A., Grogan G., Burley G. A. (2024). Gram-Scale
Enzymatic Synthesis of 2′-Deoxyribonucleoside Analogues Using
Nucleoside Transglycosylase-2. Chem. Sci..

[73] Salihovic A., Ascham A., Rosenqvist P. S., Taladriz-Sender A., Hoskisson P. A., Hodgson D. R. W., Grogan G., Burley G. A. (2025). Biocatalytic
Synthesis of Ribonucleoside Analogues Using Nucleoside Transglycosylase-2. Chem. Sci..

[74] Parkin D. W., Horenstein B. A., Abdulah D. R., Estupiñán B., Schramm V. L. (1991). Nucleoside
Hydrolase from *Crithidia fasciculata*: Metabolic Role,
Purification, Specificity, and Kinetic Mechanism. J. Biol. Chem..

[75] Horenstein B. A., Parkin D. W., Estupiñán B., Schramm V. L. (1991). Transition-State
Analysis of Nucleoside Hydrolase from *Crithida fasciculata*. Biochemistry.

[76] Capon B. (1969). Mechanism in Carbohydrate Chemistry. Chem. Rev..

[77] Horenstein B. A., Schramm V. L. (1993). Electronic Nature
of the Transition State for Nucleoside
Hydrolase. A Blueprint for Inhibitor Design. Biochemistry.

[78] Horenstein B. A., Schramm V. L. (1993). Correlation of the
Molecular Electrostatic Potential
Surface of an Enzymatic Transition State with Novel Transiton-State
Inhibitors. Biochemistry.

[79] Degano M., Almo S. C., Sacchettini J. C., Schramm V. L. (1998). Trypanosomal Nucleoside
Hydrolase. A Novel Mechanism from the Structure with a Transition
State Inhibitor. Biochemistry.

[80] Versées W., Decanniere K., Van Holsbeke E., Devroede N., Steyaert J. (2002). Enzyme-Substrate
Interactions in the Purine-Specific Nucleoside Hydrolase from *Trypanosoma vivax*. J. Biol. Chem..

[81] Versées W., Barlow J., Steyaert J. (2006). Transition-State Complex
of the Purine-Specific
Nucleoside Hydrolase of *T. vivax*: Enzyme Conformational
Changes and Implications for Catalysis. J. Mol.
Biol..

[82] Loverix S., Geerlings P., McNaughton M., Augustyns K., Vandemeulebroucke A., Steyaert J., Versées W. (2005). Substrate-Assisted
Leaving Group Activation in Enzyme-Catalyzed *N*-Glycosidic
Bond Cleavage. J. Biol. Chem..

[83] Parkin D. W. (1996). Purine-Specific
Nucleoside *N*-Ribohydrolase from *Trypanosoma*
*brucei brucei*. J. Biol. Chem..

[84] Singh V., Lee J. E., Núñez S., Howell P. L., Schramm V. L. (2005). Transition
State Structure of 5′-Methylthioadenosine/S-Adenosylhomocysteine
Nucleosidase from *Escherichia coli* and Its Similarity
to Transition State Analogues. Biochemistry.

[85] Singh V., Schramm V. L. (2007). Transition-State Analysis of *S. pneumoniae* 5′-Methylthioadenosine Nucleosidase. J. Am. Chem. Soc..

[86] Namanja-Magliano H. A., Stratton C. F., Schramm V. L. (2016). Transition
State Structure and Inhibition
of Rv0091, a 5′-Deoxyadenosine/5′-Methylthioadenosine
Nucleosidase from *Mycobacterium tuberculosis*. ACS Chem. Biol..

[87] Namanja-Magliano H. A., Evans G. B., Harijan R. K., Tyler P. C., Schramm V. L. (2017). Transition
State Analog Inhibitors of 5′-Deoxyadenosine/5-Methylthioadenosine
Nucleosidase from *Mycobacterium*
*tuberculosis*. Biochem..

[88] Stoeckler J. D., Cambor C., Kuhns V., Shih-Hsi C., Parks R. E. (1982). Inhibitors
of Purine Nucleoside Phosphorylase. Biochem.
Pharmacol..

[89] Stoeckler J. D., Cambor C., Parks R. E. (1980). Human Erythrocytic
Purine Nucleoside
Phosphorylase: Reaction with Sugar-Modified Nucleoside Substrates. Biochemistry.

[90] Kline P. C., Schramm V. L. (1995). Pre-Steady-State
Transition-State Analysis of the Hydroytic
Reaction Catalyzed by Purine Nucleoside Phosphorylase. Biochemistry.

[91] Fedorov A., Shi W., Kicska G., Fedorov E., Tyler P. C., Furneaux R. H., Hanson J. C., Gainsford G. J., Larese J. Z., Schramm V. L., Almo S. C. (2001). Transition
State Structure of Purine Nucleoside Phosphorylase
and Principles of Atomic Motion in Enzymatic Catalysis. Biochemistry.

[92] Murkin A. S., Birck M. R., Rinaldo-Matthis A., Shi W., Taylor E. A., Schramm V. L. (2007). Neighboring Group Participation in
the Transition State
of Human Purine Nucleoside Phosphorylase. Biochemistry.

[93] Schramm V. L., Schwartz S. D. (2018). Promoting Vibrations and the Function of Enzymes. Emerging
Theoretical and Experimental Convergence. Biochemistry.

[94] Marshall P., Reed C. G., Sinnott M. L., Souchard I. J. L. (1977). Role of the Substituent
at C5 of the Pyranose Ring in Catalysis by *E. coli* (*LacZ*) β-Galactosidase. J. Chem. Soc., Perkin Trans..

[95] McCarter J. D., Adam M. J., Withers S. G. (1992). Binding Energy and Catalysis: Fluorinated
and Deoxygenated Glycosides as Mechanistic Probes of *Escherichia
coli* (*LacZ*) β-Galactosidase. Biochem. J..

[96] Rivera-Sagredo A., Cañada F. J., Nieto O., Jiménez-Barbero J., Martín-Lomas M. (1992). Substrate
Specificity of Small Intestine Lactase. Assessment
of the Role of the Substrate Hydroxyl Groups. Eur. J. Biochem..

[97] Namchuk M. N., Withers S. G. (1995). Mechanism of *Agrobacterium* β-Glucosidase:
Kinetic Analysis of the Role of Noncovalent Enzyme/Substrate Interactions. Biochemistry.

[98] Mega T., Matsushima Y. (1983). Energy of
Binding of *Aspergillus*
*oryzae* β-Glucosidase
with the Substrate, and the Mechanism
of Its Enzymic Action. J. Biochem..

[99] Bock K., Adelhorst K. (1990). Derivatives
of Methyl β-Lactoside as Substrates
for and Inhibitors of β-D-Galactosidase from *E. coli*. Carbohydr. Res..

[100] Roth N. J., Huber R. E. (1996). The β-Galactosidase (*Escherichia coli*) Reaction Is Partly Faciliatated by Interactions
of His-540 with the C6 Hydroxyl of Galactose. J. Biol. Chem..

[101] Frandsen T. P., Stoffer B. B., Palcic M. M., Hof S., Svensson B. (1996). Structure and Energetics of the Glucoamylase-Isomaltose
Transition-State Complex Probed by Using Modelling and Deoxygenated
Substrates Coupled with Site Directed Mutagenesis. J. Mol. Biol..

[102] Hakamata W., Nishio T., Oku T. (2000). Hydrolytic
Activity
of α-Galactosidases against Deoxy Derivatives of *p*-Nitrophenyl α-D-Galactopyranoside. Carbohydr.
Res..

[103] Nidetzky B., Eis C., Albert M. (2000). Role of Non-Covalent
Enzyme-Substrate Interactions in the Reaction Catalyzed by Cellobiose
Phosphorylase from *Cellulomonas uda*. Biochem. J..

[104] Xu J., McRae M. A. A., Harron S., Rob B., Huber R. E. (2004). A Study
of the Relationships of Interactions between Asp-201, Na^+^ or K^+^, and Galactosyl C6 Hydroxyl and Their Effects on
Binding and Reactivity of β-Galactosidase. Biochem. Cell Biol..

