# Supplementary material for: Commonality of Mechanism in Glycoside Hydrolases, Nucleoside Hydrolases, and Phosphorylases: Importance of Side-Chain Conformation Preorganization
Source: JACS Au. 2025 Nov 7;5(11):5568–77. doi: 10.1021/jacsau.5c01069 (PMC12648331; doi:10.1021/jacsau.5c01069)
Supplement: Supplementary file 1 [file au5c01069_si_001.pdf]

Supporting Information for

Commonality of Mechanism in Glycoside Hydrolases, Nucleoside Hydrolases and Phosphorylases: Importance of Side Chain Conformation Preorganization

Po-Sen Tseng,[a,b,c] Jonathan C. K. Quirke,[a,b,c] W. Jonathan Lin[a,b,c] and David Crich[a,b,c,*]

a) Department of Pharmaceutical and Biomedical Sciences, University of Georgia, 250 West Green Street, Athens, GA 30602, USA

b) Department of Chemistry, University of Georgia, 302 East Campus Road, Athens, GA 30602, USA,

c) Complex Carbohydrate Research Center, University of Georgia, 315 Riverbend Road, Athens, GA 30602, USA

Additional Information on Privateer Analysis: Wherever possible, Privateer analysis was carried out to remove poor quality structures. Structures that led to an RSCC of below 0.8, structures that repeatedly led to failure in analysis, and structures missing a .mtz file were omitted. Cases that registered as potentially having an incorrect ring conformation were still included in the analysis, as higher energy conformations in the -1 subsite would not be considered irregular. Structures containing simple mono- or oligosaccharides were run using the Privateer program's website (https://privateer.york.ac.uk/), while structures containing substituted sugars not found on the Chemical Component Directory were validated using the downloadable Privateer program CCP4i2 (version 9.0.009).

Table 1. Side chain and ring conformations of furanoside ligands bound to L-arabinose processing enzymes

Enzyme Function	*gg*	*gt*	*tg*	Ring conformation	Total	H-Bonding to side chain
α-L-Arabinofuranosidase	19	0	0	E_4/E_2	19	16
β-L-Arabinofuranosidase	10	0	0	$E_4/{}^3T_2$	10	9

Table 2. Side chain and ring conformations of furanoside ligands bound to D-fructose processing enzymes

	Side chain conformation			***Anomeric side chain conformation***					
**Enzyme Function**	*gg*	*gt*	*tg*	*gg*	*gt*	*tg*	**Ring conformation**	**Total**	**H-Bonding to side chain**
β-D-Fructofuranosidase	0	28	0	1	26	1	E_3	28	27
β-D-Transfructofuranosidase	1	11	0	4	8	0	$E_3/{}^4T_3$	13[a]	7
α-D-Arabinofuranosidase/ α-D-Fructofuranosidase	2	0	0	0	0	1	${}^3T_4/E_5$[b]	2	2

[a]One structure has an ambiguous side chain conformation.

[b] The arabinofuranosyl substrate has the ${}^3T_4$ ring conformation, while the fructofuranosyl substrate has the E_5 ring conformation.

Table 3. Ligand side chain and ring conformations in complexes with D-nucleoside processing enzymes

Enzyme Function	*gg*	*gt*	*tg*	Ring conformation	Total	H-Bonding to side chain
D-Nucleoside Hydrolase (NH)	0	19	0	^4E	19	19
D-Nucleoside Phosphorylase (NP)	5	83	1	^4E	89	81
D-Nucleoside 2'-Deoxyribosyl-transferase (NDT)	0	6	0	E_O	6	6
D-5'-Methylthioadenosine/ D-*S*-Adenosylhomocysteine Nucleosidase (MTAN)	1	3	41	$^2T_3$/^4E	45	4
D-5'-Methylthioadenosine Phosphorylase (MTAP)	0	21	0	^4E/^OE	21	1

Table S1. Raw data collected from the PDB for arabinofuranosidases

Ligand ID	Ligand Residue	PDB ID	Res. (Å)	GH Family	Enzyme Function	Ring Conformation	Side Chain Conformation	H-bonding to O5	RSCC
FUB	403A-B	5GLQ	1.70	43	α-L-Arabino-furanosidase	$^{3}T_{2}$	*gg*	Asp57 (=O, OH), Arg346 (=N, NH) or Asp57 (OH), Arg346 (=N, NH)	0.89-0.93
EDG	601A-B	5JOX	1.80	43	α-L-Arabino-furanosidase	E_4	*gg*	Arg283 (=N, NH), Asp34 (=O, OH)	-
AHR	D2	1QW8	1.80	51	α-L-Arabino-furanosidase	E_4	*gg*	Tyr246 (OH), Gln351 (NH)	0.78, 0.79
AHR	3D-F	2VRQ	2.00	51	α-L-Arabino-furanosidase	E_4	*gg*	Tyr242 (OH), Gln347 (NH)	0.89-0.91
AHR	550A-B	1PZ2	2.00	51	α-L-Arabino-furanosidase	E_2	*gg*	Tyr246 (OH), Gln351 (NH)	0.68-0.70
AHR	488D, 487F	3UG4	2.15	51	α-L-Arabino-furanosidase	E_4	*gg*	Tyr257 (OH), Gln346 (NH)	0.80-0.81
AHR	710A	6ZPY	1.27	51	α-L-Arabino-furanosidase	E_4	*gg*	Tyr402 (OH)	0.87
EDG	701AAA	6ZQ1	1.70	51	α-L-Arabino-furanosidase	E_4	*gg*	Tyr402 (OH)	-
KHP	1750A, 1751B	1QW9	1.20	51	α-L-Arabino-furanosidase	E_4	*gg*	Tyr246 (OH), Gln351 (NH)	0.88-0.89 (Using program)
LXE	601A-B	6SXV	1.40	51	α-L-Arabino-furanosidase	E_2	*gg*	Tyr246 (OH), Gln351 (NH)	-
LX5	601AAA-BBB	6SXU	1.40	51	α-L-Arabino-furanosidase	^{1}E	*gg*	Gln351 (NH) or 0	-
LX5	711AAA	6ZPZ	1.71	51	α-L-Arabino-furanosidase	E_2	*gg*	0*	-

LXE	711AAA	6ZQ0	1.54	51	α-L-Arabino-furanosidase	E_2	*gg*	0*	-
AHR	601A	1WD4	2.07	54	α-L-Arabino-furanosidase	^3E	*gg*	Asn223 (backbone =O, backbone NH), Asp219 (=O)	0.84
LX5	510AAA	6SXS	1.86	54	α-L-Arabino-furanosidase	E_2	*gg*	Asn223 (backbone =O)	-
LXE	507A	6SXT	1.44	54	α-L-Arabino-furanosidase	E_2	*gg*	0*	-
AHR	402A	4O8O	1.21	62	α-L-Arabino-furanosidase	E_4	*gg*	Lys87 (NH), Asp88 (OH)	0.92
EDG	401A-B	6F1J	1.25	62	α-L-Arabino-furanosidase	E_4	*gg*	Asp52 (OH)	-
FUB	503A	3WN0	1.90	62	α-L-Arabino-furanosidase	E_2	*gg*	Lys201 (NH), Asp202 (OH)	0.92
FE0	702A	7BZL	2.30	127	β-L-Arabino-furanosidase	E_4	*gg*	His142 (NH)	-
FE0	702A	7DIF	1.75	127	β-L-Arabino-furanosidase	E_4	*gg*	His142 (NH)	-
FUB	701A	3WKX	2.00	127	β-L-Arabino-furanosidase	$^3T_2$	*gg*	His142 (NH)	0.84
FUB	701A	3WRG	2.23	127	β-L-Arabino-furanosidase	$^3T_2$	*gg*	His142 (NH)	0.93
07Y	702C ASM 1-2	7EXU	2.30	127	β-L-Arabino-furanosidase	$^3T_2$	*gg*	His142 (NH)	0.84
09X	801B ASM 1-2	7EXW	2.20	127	β-L-Arabino-furanosidase	E_2	*gg*	His142 (NH)	0.88
UI5	702C	8QF2	2.35	127	β-L-Arabino-furanosidase	E_4	*gg*	His142 (NH)	-

FUB	6B	5MUI	1.77	137	β-L-Arabino-furanosidase	^{3}E	*gg*	Asp273 (=O, OH), Cys62 (SH), Lys334 (NH)	0.88
PJ5	1204AAA	6YQH	1.41	146	β-L-Arabino-furanosidase	E_2	*gg*	Gln686 (NH), Gln687 (NH), Glu318 (=O)	-
UHU/UI5	901E/901C	8QF8	2.40	146	β-L-Arabino-furanosidase	E_4	*gg*	0	-
BXX	602A, 602E	7V1W	1.86	172	α-D-Arabino-furanosidase	$^{3}T_4$	*gg*	Ala297 (NH), Asp292 (OH)	0.80-0.81

*The side chain hydroxyl group is close to other H-bond donors in the active site, but does not register as H-bonding with the enzyme in the PDB structure viewer

Table S2. Raw data collected from the PDB for fructofuranosidases

Ligand ID	Ligand Residue	PDB ID	Res. (Å)	GH Family	Enzyme Function	Ring Conformation	Side Chain Conformation (+ C1 Chain)	H-bonding to O6	RSCC
FRU	1000A-B	6FJG	1.73	32	β-D-Fructofuranosidase	E_3	*gt* (+*gt*)	Gln97 (=O), Asn79 (NH), Trp105 (NH)	0.93
9OK	2000A-B	5O47	1.91	32	β-D-Fructofuranosidase	E_3	*gt* (+*gt*)	Gln97 (=O), Asn79 (NH), Trp105 (NH)	0.84 (Using program)
FRU	801A	1Y9G	1.87	32	β-D-Fructofuranosidase	E_3	*gt* (+*gt*)	Gln57 (NH), Asn40 (NH), Trp65 (NH)	0.87
FRU	2D	2ADD	2.50	32	β-D-Fructofuranosidase	$^4T_3$	*gt* (+*tg*)	Asn21 (NH), Gln38 (=O)	0.92
FRU	527A-B	3PIJ	1.80	32	β-D-Fructofuranosidase	E_3	*gt* (+*gt*)	Met81 (S), Gln70 (=O), Asn53 (NH), Trp78 (NH)	0.90-0.91
FRU	6C-D	3U14	2.24	32	β-D-Fructofuranosidase	E_3	*gt* (+*gg*)	Asn49 (NH), Gln68 (NH), Trp76 (NH)	0.84-0.85
FRU	2I, 2K	5FIX	2.01	32	β-D-Fructofuranosidase	E_3	*gt* (+*gt*)	Gln97 (=O), Asn79 (NH), Trp105 (NH)	0.93-0.95
FRU	2C, 2G	5FK7	2.05	32	β-D-Fructofuranosidase	E_3	*gt* (+*gt*)	Gln97 (=O), Asn79 (NH), Trp105 (NH)	0.92-0.94
FRU	3C, 3G	5FK8	1.88	32	β-D-Fructofuranosidase	E_3	*gt* (+*gt*)	Gln97 (=O), Asn79 (NH), Trp105 (NH)	0.92-0.94
FRU	3A-B	5FKB	1.78	32	β-D-Fructofuranosidase	E_3	*gt* (+*gt*)	Gln97 (=O), Asn79 (NH), Trp105 (NH)	0.92-0.94

FRU	1F, 1J	5FKC	1.82	32	β-D-Fructofuranosidase	E_3	*gt* (+*gt*)	Gln97 (=O), Asn79 (NH), Trp105 (NH)	0.93-0.95
FRU	1666A-B	5FMB	1.91	32	β-D-Fructofuranosidase	E_3	*gt* (+*gt*)	Gln97 (=O), Asn79 (NH), Trp105 (NH)	0.92-0.95
FRU	1000A-B	5FMC	1.84	32	β-D-Fructofuranosidase	E_3	*gt* (+*gt*)	Gln97 (=O), Asn79 (NH), Trp105 (NH)	0.93-0.95
FRU	2000A-B	5NSL	1.70	32	β-D-Fructofuranosidase	E_3	*gt* (+*gt*)	Gln97 (=O), Asn79 (NH), Trp105 (NH)	0.92-0.93
FRU	2B	5XHA	2.10	32	β-D-Fructofuranosidase	E_3	*gt* (+*gt*)	0	0.86
FRU	1000A-B	6FJE	1.85	32	β-D-Fructofuranosidase	E_3	*gt* (+*gt*)	Gln97 (=O), Asn79 (NH), Trp105 (NH)	0.90-0.91
FRU	501A	6NU8	1.80	32	β-D-Fructofuranosidase	E_3	*gt* (+*gt*)	Gln63 (=O), Asn46 (NH), Lys74 (NH)	0.87
FRU	606A	6NUN	1.87	32	β-D-Fructofuranosidase	E_3	*gt* (+*gt*)	Met81 (S), Gln70 (=O), Asn53 (NH), Trp78 (NH)	0.94
FRU	1001A-B	6S2G	2.03	32	β-D-Fructofuranosidase	E_3	*gt* (+*gt*)	Gln97 (=O), Asn79 (NH), Trp105 (NH)	0.92-0.94
FRU	1001A-B	6S2H	1.80	32	β-D-Fructofuranosidase	E_3	*gt* (+*gt*)	Gln97 (=O), Asn79 (NH), Trp105 (NH)	0.94-0.95
FRU	1000A-B	6S3Z	1.85	32	β-D-Fructofuranosidase	E_3	*gt* (+*gt*)	Gln97 (=O), Asn79 (NH), Trp105 (NH)	0.91-0.92
FRU	2B	7BWC	1.95	32	β-D-Fructofuranosidase	E_3	*gt* (+*gt*)	Trp87 (NH), Asn62 (NH), Met90 (S), Gln79 (NH)	0.88

FRU	4I-J	5FMD	1.78	32	β-D-Fructofuranosidase	E_3	*gt* (+*gt*)	Gln97 (=O), Asn79 (NH), Trp105 (NH)	0.92-0.93
FRU	500A-B	7VCP	2.00	32	β-D-Fructofuranosidase	E_3	*gt* (+*gt*)	Trp62 (NH), Asn39 (NH), Gln56 (=O)	0.90
FRU	714A, 710B, 714C, 712D	8BES	1.86	32	β-D-Fructofuranosidase	E_3	*gt* (+*gt*)	Asn187 (NH), Trp448 (NH) **	0.85-0.86
FRU	2L-O	8BET	2.38	32	β-D-Fructofuranosidase	E_3	*gt* (+*gt*)	Asn187 (NH), Gln205 (=O)	0.84-0.88
FRU	1G, 1J, 1L	8BEU	2.27	32	β-D-Fructofuranosidase	E_3	*gt* (+*gt*)	Asn187 (NH), Gln205 (=O), Gln216 (NH, 1J and 1L only)	0.88-0.89
FRU	2G, 2J, 2L	1W2T	1.87	32	β-D-Fructofuranosidase	E_3	*gt* (+*gt*)	Gln33 (=O), Asn16 (NH), Trp41 (NH)	0.84
FRU	2B	3LDK	2.20	32	β-D-Transfructo-furanosidase	$^4T_3$	*gt* (+*gt*)	0	0.90
FRU	3B	3LDR	2.10	32	β-D-Transfructo-furanosidase	$^4T_3$	*gt* (+*gt*)	0	0.88
FRU	4B	3LEM	2.10	32	β-D-Transfructo-furanosidase	$^4T_3$	*gt* (+*gt*)	0	0.90
FRU	1B	3LIH	2.20	32	β-D-Transfructo-furanosidase	E_3	*gt* (+*gt*)	0	0.91
FRU	3B	2YFT	1.85	68	β-D-Transfructo-furanosidase	E_3	*gt* (+*gg*)	Trp128 (NH), Met153 (S)	0.93
FRU	2B	1PT2	2.10	68	β-D-Transfructo-furanosidase	E_3	*gt* (+*gt*)	Trp85 (NH)	0.91
FRU	1B	3BYN	2.10	68	β-D-Transfructo-furanosidase	E_3	*gt* (+*gt*)	Trp85 (NH)	0.91
FRU	6C	6VHQ	2.05	68	β-D-Transfructo-furanosidase	E_3	*gt* (+*gg*)	Trp85 (NH)	0.80

FRU	2B	7FDZ	1.35	68	β-D-Transfructo-furanosidase	E_3	*gt* (+*gt*)	Trp67 (NH), His119 (NH)	0.85
FRU	3E	9J4I	1.96	91	β-D-Transfructo-furanosidase	E_3	*gt* (+*gg*)	H bonds to O1	0.82
9F3	501A-F	5ZKU	2.32	91	β-D-Transfructo-furanosidase	$^4T_3$	*gt* (+*gg*)	Asp199 (=O, OH) or 0	0.82-0.87 (Using program)
9F3	501B	5ZLA	1.70	91	β-D-Transfructo-furanosidase	E_5	*gg* (+*gt*)	Asp199 (=O)	0.81 (Using program)
FRU	3F, 3H	8HUI	1.44	91	β-D-Transfructo-furanosidase	3F: E_5<br>3H: E_3	3F: *gg* (+*tg*)<br>3H: *gt* (+*gg*)	0	0.93
FRU	702H, 702R	7V1X	1.76	172	α-D-Fructofuranosidase	E_5	*gg* (+*tg*)	Trp298 (NH), Asp292 (OH)	0.80-0.81

**Fructose flips in the active site so that C1-C2 bond is in the C5-C6 side chain position; both side chains are *gt*

Table S3. Side chain conformation distribution across PDB for arabino- and fructofuranosidases

GH Family	Enzyme Function	*gg*	*gt*	*tg*	Ambig	H-bonding to O5/O6
32	β-D-Fructofuranosidase or β-D-Transfructofuranosidase		32			27
43	α-L-Arabinofuranosidase	2				2
51	α-L-Arabinofuranosidase	11				9
54	α-L-Arabinofuranosidase	3				2
62	α-L-Arabinofuranosidase	3				3
68	β-D-Transfructofuranosidase		5			5
91	β-D-Transfructofuranosidase	1	2		1	2
127	β-L-Arabinofuranosidase	7				7
137	β-L-Arabinofuranosidase	1				1
146	β-L-Arabinofuranosidase	2				1
172	α-D-Arabinofuranosidase/α-D-Fructofuranosidase	2				2

Table S4. PDB structures with ligands bound in a catalytically inactive orientation

Ligand ID	Ligand Residue	PDB ID	Res. (Å)	GH Family	Enzyme Function	Conformation	H-bonding to O5/O6	RSCC
AHR	901A	5OPJ	2.05	146	β-L-Arabinofuranosidase	*gg*	Glu215 (=O)	0.93
FRU	603D	8I2R	1.36	68	β-D-Transfructofuranosidase	*gg*	Trp106 (NH)	0.92
FRU	604A	6M0E	1.35	68	β-D-Transfructofuranosidase	*gg*	Trp106 (NH)	0.93

Table S5. Raw data collected for NHs

Ligand ID	Ligand Residue	PDB ID	Res. (Å)	NH Family	Enzyme Function	Ring Conformation	Side Chain Conformation	H-bonding to O5	RSCC
NOS	802B	1KIC	1.60	IAG-NH	Purine Nucleosidase	^{4}E	*gt*	Asn173 (NH), Glu184 (=O)	0.78
AD3	802B	1KIE	2.00	IAG-NH	Purine Nucleosidase	E_{O}	*gt*	Asn173 (NH), Glu184 (=O)	0.71
IMH	401A, 402B	2FF1	2.07	IAG-NH	Purine Nucleosidase	^{4}E	*gt*	Asn173 (NH), Glu184 (=O)	-
IMH	501A, 502B	2FF2	2.20	IAG-NH	Purine Nucleosidase	^{4}E	*gt*	Asn173 (NH), Glu184 (=O)	-
PIR	400A-D	2MAS	2.30	IU-NH	Purine Nucleosidase	^{4}E	*gt*	Asn160 (NH), Glu166 (OH)	-
IMH	501A, 502B	3B9G	1.40	IAG-NH	Purine Nucleosidase	^{4}E	*gt*	Asn173 (NH), Glu184 (OH)	-
JMQ	1002A, 1003B	3EPW	1.30	IAG-NH	Purine Nucleosidase	$^{1}T_{\mathrm{N}}$	*gt*	Asn173 (NH), Glu184 (OH)	-
IMQ	1002A, 1003B	3EPX	1.85	IAG-NH	Purine Nucleosidase	$^{1}T_{\mathrm{N}}$	*gt*	Asn173 (NH), Glu184 (OH)	-
AGV	401A	4I71	1.28	IAG-NH	Purine Nucleosidase	E_{N}	*gt*	Asn173 (NH), Glu184 (OH)	-
UA2	402A-B	4I72	2.05	IAG-NH	Purine Nucleosidase	$^{4}T_{3}$ or ^{4}E	*gt*	Asn173 (NH), Glu184 (OH)	-
MBY	402A-D	4I73	2.18	IAG-NH	Purine Nucleosidase	E_{N}	*gt*	Asn173 (NH), Glu184 (OH)	-
MBY	408A	4I74	1.68	IAG-NH	Purine Nucleosidase	E_{N}	*gt*	Asn173 (NH), Glu184 (OH)	-
BDR	402A	5TSQ	1.53	IU-NH	Purine Nucleosidase	^{2}E	*gt*	Asn160 (NH), Glu166 (OH)	0.92
IMH	402A-D	6ZK2	2.20	-	Uridine Nucleosidase	E_{N}	*gt*	Asn168 (NH), Glu174 (OH)	-
IMH	402A, 401B	6ZK5	1.90	-	Uridine Nucleosidase	$^{4}T_{\mathrm{N}}$	*gt*	Asn158 (NH), Glu164 (OH)	-
RIB	2001A	1YOE	1.78	CU-NH	Ribosylpyrimidine Nucleosidase	^{2}E or E_{1}	*gt*	Asn158 (NH), Glu164 (OH)	0.91

DNB	502A-D	3G5I	2.10	CU-NH	Ribosylpyrimidine Nucleosidase	^4E or ^4T_N	*gt*	Asn158 (NH), Glu164 (OH)	-
DNB	502A-D	3MKN	2.00	CU-NH	Ribosylpyrimidine Nucleosidase	^4E	*gt*	Asn158 (NH), Glu164 (OH)	-
URI	802A	5ILW	1.98	-	rRNA *N*-glycosylase	^3E	*gt*	Tyr111 (backbone NH), Glu112 (=O)	0.87

Table S6. Raw data collected for NPs

Ligand ID	Ligand Residue	PDB ID	Res. (Å)	NP Family	Enzyme Function	Ring Conformation	Side Chain Conformation	H-bonding to O5	RSCC
IMH	301A, 302B, 303C, 304D, 305E, 306F	1NW4	2.20	NP-I	Purine Nucleoside Phosphorylase	$^{4}T_{3}$	*gt*	His7 (NH)	-
MDR	245A, 246B, 247C	1OVG	2.20	NP-I	Purine Nucleoside Phosphorylase	$^{3}T_{2}$ or E_{O} or E_{2}	*gt*	His4 (NH)	0.80-0.88
2FA	306A, 307B, 308C	1PK9	1.90	NP-I	Purine Nucleoside Phosphorylase	^{4}E or $^{4}T_{O}$	*gt*	His4 (NH), Met64 (S) or His4 (NH)	0.93-0.94
2FD	446A, 447B, 448C	1PKE	2.30	NP-I	Purine Nucleoside Phosphorylase	^{4}E	*gt*	His4 (NH), Met64 (S) or His4 (NH)	0.93-0.95
NOS	1245A, 1246B, 1247C	1PR0	2.20	NP-I	Purine Nucleoside Phosphorylase	^{4}E	*gt*	His4 (NH), Met64 (S) or His4 (NH)	0.83-0.89
FMB	246B, 247C	1PR1	2.30	NP-I	Purine Nucleoside Phosphorylase	^{4}E	*gt*	His4 (NH) or 0	0.88-0.90
MDR	1245A, 1246B	1PR2	2.30	NP-I	Purine Nucleoside Phosphorylase	E_{O}	*gt*	His4 (NH)	0.83-0.84
MTP	1245A, 1246B, 1247C	1PR4	2.40	NP-I	Purine Nucleoside Phosphorylase	^{4}E	*gt*	His4 (NH), Met64 (S)	0.91-0.92
TBN	1245A, 1246B, 1247C	1PR5	2.50	NP-I	Purine Nucleoside Phosphorylase	^{4}E	*gt*	His4 (NH)	0.82-0.92
XYA	1247C	1PR6	2.10	NP-I	Purine Nucleoside Phosphorylase	E_{3}	*gt*	His4 (NH), Met64 (S)	0.89
MTI	301A, 302B, 303C, 304D, 305E, 306F	1Q1G	2.02	NP-I	Purine Nucleoside Phosphorylase	^{4}E	*tg*	0	-

IMH	300A	1RR6	2.50	NP-I	Purine Nucleoside Phosphorylase	E_3	*gt*	His257 (NH)	-
DIH	300A	1RSZ	2.20	NP-I	Purine Nucleoside Phosphorylase	^{2}E	*gt*	His257 (NH)	-
ADN	252A-F	1VHW	1.54	NP-I	Purine Nucleoside Phosphorylase	E_O	*gt*	His5 (NH)	0.93-0.94
2FD	300A	1Z34	2.40	NP-I	Purine Nucleoside Phosphorylase	^{4}E	*gt*	His4 (NH), Met64 (S)	0.89
2FA	300A	1Z35	2.50	NP-I	Purine Nucleoside Phosphorylase	^{4}E	*gt*	His4 (NH)	0.89
NOS	300A	1Z38	2.50	NP-I	Purine Nucleoside Phosphorylase	$^{4}T_O$	*gt*	His4 (NH)	0.90
DIH	300A	2A0W	2.28	NP-I	Purine Nucleoside Phosphorylase	^{2}E	*gt*	0	-
DIH	300A	2A0X	2.28	NP-I	Purine Nucleoside Phosphorylase	^{2}E	*gt*	0	-
DIH	300A	2A0Y	2.28	NP-I	Purine Nucleoside Phosphorylase	^{2}E	*gt*	Asp257 (=O)	-
ADN	1216A, 1215B	2AC7	1.70	NP-I	Purine Nucleoside Phosphorylase	$^{4}T_3$	*gt*	Met64 (S)	0.94
P1G	293A	2AI1	2.00	NP-I	Purine Nucleoside Phosphorylase	E_O	*gt*	His257 (NH)	0.92
P1D	293A	2AI2	1.70	NP-I	Purine Nucleoside Phosphorylase	E_O	*gt*	His257 (NH)	0.92
P2G	293A	2AI3	1.70	NP-I	Purine Nucleoside Phosphorylase	$^{O}T_4$	*gt*	His257 (NH)	0.85
NOS	1248A	2BSX	2.00	NP-I	Purine Nucleoside Phosphorylase	E_O	*gt*	His7 (NH)	0.85
DIH	401A, 403B, 402C	2P4S	2.20	NP-I	Purine Nucleoside Phosphorylase	^{2}E	*gt*	His342 (NH)	-
R1X	302A, 302C, 302E	3ENZ	2.03	NP-I	Purine Nucleoside Phosphorylase	E_3	*gt*	His7 (NH)	0.80
ADN	300A, 301B, 302C	3F8W	2.30	NP-I	Purine Nucleoside Phosphorylase	^{4}E	*gt*	Tyr202 (OH), His259 (NH)	0.92-0.95
NOS	301A-C	3FAZ	1.90	NP-I	Purine Nucleoside Phosphorylase	^{4}E or $^{2}T_3$	*gt*	Tyr202 (OH), His259 (NH)	0.83-0.93

R1P	301A, 302B, 300C	3FB1	2.00	NP-I	Purine Nucleoside Phosphorylase	$^{2}T_{3}$ or $^{2}T_{1}$	*gt*	Tyr202 (OH), His259 (NH) or Tyr202 (OH)	0.93
GMP	300A, 301C	3IEX	2.05	NP-I	Purine Nucleoside Phosphorylase	$^{4}T_{3}$ or ^{4}E	*gt*	Tyr202 (OH), His259 (NH)	0.89-0.91
IMH	280A-B	3MB8	1.90	NP-I	Purine Nucleoside Phosphorylase	^{4}E	*gt*	His12 (NH)	-
DIH	500A-F	3OCC	1.70	NP-I	Purine Nucleoside Phosphorylase	E_{3}	*gt*	His5 (NH)	-
DIH	500A-L	3OF3	1.83	NP-I	Purine Nucleoside Phosphorylase	^{2}E	*gt*	His18 (NH)	-
IM5	290E, 290Q, 291S-T, 290U, 292Y	3PHB	2.30	NP-I	Purine Nucleoside Phosphorylase	^{2}E or $^{2}T_{N}$ or E_{N}	*gt*	His257 (NH)	-
IM5	501A-F	3PHC	2.00	NP-I	Purine Nucleoside Phosphorylase	^{2}E	*gt*	His7 (NH)	-
ADN	251A, 251C-D, 251F	3U40	2.05	NP-I	Purine Nucleoside Phosphorylase	E_{O} or ^{4}E	*gt*	His9 (NH), Met69 (S) or His9 (NH)	0.87-0.92
ADN	236A	3UAW	1.20	NP-I	Purine Nucleoside Phosphorylase	^{4}E	*gt*	His4 (NH), Met64 (S)	0.96
NOS	236A	3UAX	1.20	NP-I	Purine Nucleoside Phosphorylase	^{4}E	*gt*	His4 (NH), Met64 (S)	0.95
ADN	236A	3UAY	1.40	NP-I	Purine Nucleoside Phosphorylase	^{4}E	*gt*	His4 (NH), Met64 (S)	0.96
NOS	236A	3UAZ	1.40	NP-I	Purine Nucleoside Phosphorylase	^{4}E	*gt*	His4 (NH), Met64 (S)	0.95
FMC	300A-C	3UT6	1.90	NP-I	Purine Nucleoside Phosphorylase	^{4}E or E_{3}	*gt*	His4 (NH)	0.93-0.95
6CR	301A	4DAE	2.35	NP-I	Purine Nucleoside Phosphorylase	E_{3}	*gt*	His4 (NH), Met64 (S)	0.91
IM5	301A-C	4EAR	1.70	NP-I	Purine Nucleoside Phosphorylase	$^{2}T_{N}$	*gt*	0	-

IM5	302A-C	4EB8	2.30	NP-I	Purine Nucleoside Phosphorylase	$^{2}T_{N}$	*gt*	0	-
FMC	300A, 302B	4TS9	1.77	NP-I	Purine Nucleoside Phosphorylase	$^{4}T_{3}$	*gt*	His4 (NH)	0.84-0.93
FMC	301C, 302D	4TTA	2.00	NP-I	Purine Nucleoside Phosphorylase	$^{2}T_{3}$ or E_{3}	*gt*	His4 (NH)	0.82-0.83
FMC	300A, 301C, 302D, 301F	4TTI	1.89	NP-I	Purine Nucleoside Phosphorylase	E_{3}	*gt*	His4 (NH)	0.81-0.83
FMC	302A-B, 302D	4TTJ	1.87	NP-I	Purine Nucleoside Phosphorylase	^{4}E	*gt*	His4 (NH), Met64 (S) or His4 (NH)	0.85-0.89
IM5	301A-E, 302F	5ETJ	2.30	NP-I	Purine Nucleoside Phosphorylase	^{2}E	*gt*	His257 (NH)	-
R1P	302A	5KO6	1.42	NP-I	Purine Nucleoside Phosphorylase	^{2}E	*gt*	Tyr202 (OH), His259 (NH)	0.86
CTN	301A	5TBT	2.10	NP-I	Purine Nucleoside Phosphorylase	^{4}E	*gt*	Tyr202 (OH), His259 (NH)	0.84
TBN	301A	5TBV	1.95	NP-I	Purine Nucleoside Phosphorylase	^{4}E	*gt*	Tyr202 (OH), His259 (NH)	0.93
IM5	308A, 306B, 305C, 307D, 306E, 303F	5UGF	2.20	NP-I	Purine Nucleoside Phosphorylase	^{2}E	*gt*	His257 (NH)	-
IM5	302A	6AQS	1.57	NP-I	Purine Nucleoside Phosphorylase	^{2}E	*gt*	His7 (NH)	-
FMC	300A-C, 300E-F	6F4W	2.29	NP-I	Purine Nucleoside Phosphorylase	$^{1}T_{2}$ or $^{1}T_{O}$ or ^{1}E	*gt*	His4 (NH), Met64 (S) or His4 (NH)	0.87-0.92
FMC	301A-B, 302C, 301D, 302E, 301F	6F4X	1.69	NP-I	Purine Nucleoside Phosphorylase	^{1}E or $^{4}T_{3}$ or E_{O}	*gt*	His4 (NH), Met64 (S) or His4 (NH)	0.83-0.90
FMC	301B	6XZ2	1.65	NP-I	Purine Nucleoside Phosphorylase	$^{4}T_{O}$	*gt*	His4 (NH)	0.80

DIH	401A-B, 401D-E	8SWQ	1.98	NP-I	Purine Nucleoside Phosphorylase	^{4}E or ^{2}E or $^{N}T_{6}$ or $^{4}T_{6}$	*gt*	His281 (NH)	-
IM5	401A-B, 401D-E	8SWS	1.99	NP-I	Purine Nucleoside Phosphorylase	^{2}E or $^{2}T_{N}$	*gt*	His281 (NH)	-
IMH	300A-B	8SWT	1.66	NP-I	Purine Nucleoside Phosphorylase	^{4}E	*gt*	His247 (NH)	-
IMH	301A-C	8SWU	2.34	NP-I	Purine Nucleoside Phosphorylase	^{4}E or $^{4}T_{3}$	*gt*	Tyr192 (OH), His247 (NH)	-
THM	434A	3H5Q	1.94	NP-II	Pyrimidine Nucleoside Phosphorylase	$^{3}T_{2}$	*gg*	0	0.86
ADN	1237A-B, 1238C, 1237D-E, 1238F	1ODI	2.40	NP-I	Uridine Phosphorylase	E_{O}	*gt*	His5 (NH), Met65 (S) or His5 (NH)	0.89-0.95
GMP	1237A-B, 1237D-F	1ODJ	2.40	NP-I	Uridine Phosphorylase	E_{O}	*gt*	His5 (NH)	0.84-0.89
R1P	2012B, 2042I	1RXC	2.35	NP-I	Uridine Phosphorylase	$^{2}T_{1}$	*gt*	His8 (NH)	0.81-0.83
5UD	3001A, 5002B	1TGV	2.20	NP-I	Uridine Phosphorylase	E_{O}	*gt*	His8 (NH)	0.84-0.87
R1P	1254A, 2254B	1TGY	2.20	NP-I	Uridine Phosphorylase	$^{2}T_{3}$	*gt*	His8 (NH)	0.94
BJE	707B	1Y1R	2.11	NP-I	Uridine Phosphorylase	^{2}E	*gg*	His8 (NH)	0.82
ANU	7016B	1ZL2	1.85	NP-I	Uridine Phosphorylase	^{4}E	*gt*	His8 (NH)	0.90
ANU	6F	2OEC	2.19	NP-I	Uridine Phosphorylase	E_{3}	*gt*	His7 (NH)	0.82
ANU	7016B	2PGA	1.74	NP-I	Uridine Phosphorylase	$^{4}T_{3}$	*gt*	His8 (NH)	0.85
ANU	1516C-F	3C74	2.38	NP-I	Uridine Phosphorylase	E_{3} or $^{4}T_{3}$	*gt*	His8 (NH)	0.80-0.87
ANU	7016B, 7013D	3FWP	1.86	NP-I	Uridine Phosphorylase	^{4}E	*gt*	His8 (NH)	0.81-0.89
R2B	256A-F	3KVV	1.80	NP-I	Uridine Phosphorylase	E_{2} or ^{1}E	*gt*	His8 (NH)	0.81-0.87
R2B	313A-B	3KVY	2.30	NP-I	Uridine Phosphorylase	*Planar*	*gt*	His35 (NH), Met109 (S)	0.88-0.89
R1P	1254B, 1254D, 1254F, 1254I,	3QPB	1.82	NP-I	Uridine Phosphorylase	E_{3}	*gt*	His13 (NH)	0.81-0.86

	1254K, 1254Q								
THM	301A-F	4G8J	2.12	NP-I	Uridine Phosphorylase	$^{2}T_{3}$	*gg*	His7 (NH)	0.80-0.91
THM	301A-D, 301F	4LZW	1.29	NP-I	Uridine Phosphorylase	E_{3}	*gg*	His7 (NH)	0.83-0.89
THM	301A-C, 302D	4TXJ	1.66	NP-I	Uridine Phosphorylase	E_{O}	*gt*	His22 (NH)	0.87-0.90
URI	301A-F	5C80	2.24	NP-I	Uridine Phosphorylase	$^{O}T_{1}$ or ^{O}E	*gt*	His7 (NH)	0.86-0.90
CTN	303E-F	5EFO	1.63	NP-I	Uridine Phosphorylase	E_{O}	*gt*	His7 (NH)	0.81-0.85
URI	303A-B, 302C-E	5LHV	1.29	NP-I	Uridine Phosphorylase	E_{3} or $^{4}T_{O}$	*gt*	His7 (NH)	0.81-0.86
CTN	303A, 302D	5LOK	1.11	NP-I	Uridine Phosphorylase	^{4}E	*gt*	His7 (NH)	0.83-0.87
URI	301E	5M2T	1.03	NP-I	Uridine Phosphorylase	^{O}E	*gt*	His7 (NH)	0.80
R1P	402A-B, 402D	6K5H	2.50	NP-I	Uridine Phosphorylase	E_{3}	*gt*	His19 (NH)	0.90-0.92
BJE	302A, 301B, 302C, 301D, 302E, 301F	6RCA	1.34	NP-I	Uridine Phosphorylase	^{4}E	*gt*	His7 (NH)	0.91-0.96
0NP	501A	4EAD	1.50	NP-II	Thymidine Phosphorylase	^{2}E	*gg*	0	0.94
AZZ	510A	4LHM	1.52	NP-II	Thymidine Phosphorylase	$^{O}T_{1}$	*gt*	0	0.93

Table S7. Raw data collected for NDTs

Ligand ID	Ligand Residue	PDB ID	Res. (Å)	Class	Enzyme Function	Ring Conformation	Side Chain Conformation	H-bonding to O5	RSCC
V3M	202A-C	1S2D	2.10	Class I	Nucleoside 2'-Deoxyribosyltransferase	E_O	*gt*	Tyr17 (OH), Asp95 (=O, OH), Asn128 (=O)	0.84-0.92
3D1	1168A, 1170C	1S2G	2.10	Class I	Nucleoside 2'-Deoxyribosyltransferase	E_O	*gt*	Tyr17 (OH), Asp95 (=O, OH), Asn128 (=O) or Asp95 (=O, OH), Asn128 (=O)	0.80-0.85
2DR	201A-B	9F08	2.37	Class II	Nucleoside 2'-Deoxyribosyltransferase	^2E or E_3	*gt*	Asp92 (OH), Asn123 (NH)	0.87-0.89
DCZ/2DR	201A/201B	9F09	2.37	Class II	Nucleoside 2'-Deoxyribosyltransferase	$E_O/^2T_1$	*gt*	Asp92 (OH), Asn123 (NH)	0.87/0.80
RIB	202A	9GN2	2.41	Class II	Nucleoside 2'-Deoxyribosyltransferase	E_1	*gt*	Asp92 (=O, OH), Asn123 (NH)	0.80
CTN	201A	9GN4	2.48	Class II	Nucleoside 2'-Deoxyribosyltransferase	E_O	*gt*	Asp92 (OH), Asn123 (NH)	0.93

Table S8. Raw data collected for MTANs

Ligand ID	Ligand Residue	PDB ID	Res. (Å)	Enzyme Function	Ring Conformation	Side Chain Conformation	H-bonding to O5	RSCC
MTH	268A, 269B	2QTG	1.84	5'-Methylthioadenosine Nucleosidase	$^{4}T_{3}$	*tg*	0	0.88-0.93
FMC	269B	2QTT	1.93	5'-Methylthioadenosine Nucleosidase	$^{4}T_{3}$	*tg*	Met201 (S)	0.78
MTH	233A, 234B	1NC1	2.00	5'-Methylthioadenosine/ *S*-Adenosylhomocysteine Nucleosidase	^{4}E	*tg*	0	0.92
FMC	233A, 234B	1NC3	2.20	5'-Methylthioadenosine/ *S*-Adenosylhomocysteine Nucleosidase	^{4}E	*tg*	Met173 (S)	0.91
TDI	233A, 234B	1Y6Q	2.20	5'-Methylthioadenosine/ *S*-Adenosylhomocysteine Nucleosidase	E_{N}	*tg*	0	-
MTM	233A, 234B	1Y6R	2.20	5'-Methylthioadenosine/ *S*-Adenosylhomocysteine Nucleosidase	^{4}E	*tg*	0	-
SR1	233A, 235B	1Z5N	2.10	5'-Methylthioadenosine/ *S*-Adenosylhomocysteine Nucleosidase	$^{2}T_{3}$ or E_{3}	*tg*	0	0.92
MTA	5233A, 9233B	1Z5O	2.00	5'-Methylthioadenosine/ *S*-Adenosylhomocysteine Nucleosidase	$^{4}T_{3}$	*tg*	0	0.90-0.91
MTM	301A, 302B, 303C, 304D, 305E, 306F	1ZOS	1.60	5'-Methylthioadenosine/ *S*-Adenosylhomocysteine Nucleosidase	^{4}E	*tg*	0	-
FMC	229A	3BL6	1.70	5'-Methylthioadenosine/ *S*-Adenosylhomocysteine Nucleosidase	^{4}E	*tg*	Met172 (S)	0.92
DF9	233A-B	3DF9	1.95	5'-Methylthioadenosine/ *S*-Adenosylhomocysteine Nucleosidase	^{2}E	*gg*	0	-
BIG	301A, 301C	3DP9	2.30	5'-Methylthioadenosine/	^{2}E or $^{2}T_{3}$	*tg*	0	-

				S-Adenosylhomocysteine Nucleosidase				
MTM	234A-B	3EEI	1.78	5'-Methylthioadenosine/ *S*-Adenosylhomocysteine Nucleosidase	^{4}E	*tg*	0	-
FMC	501A, 502B	3NM5	1.80	5'-Methylthioadenosine/ *S*-Adenosylhomocysteine Nucleosidase	$^{4}T_{3}$ or ^{4}E	*tg*	Met174 (S)	0.91-0.94
4CT	233A, 234B	3O4V	1.75	5'-Methylthioadenosine/ *S*-Adenosylhomocysteine Nucleosidase	$^{4}T_{3}$	*tg*	0	-
MTA	1232A-B	4BMZ	1.79	5'-Methylthioadenosine/ *S*-Adenosylhomocysteine Nucleosidase	$^{4}T_{3}$ or ^{4}E	*tg*	0	0.89
2EL	301A-B	4F2P	1.64	5'-Methylthioadenosine/ *S*-Adenosylhomocysteine Nucleosidase	^{2}E or $^{2}T_{3}$	*tg*	0	-
TDI	301A-B	4F2W	2.00	5'-Methylthioadenosine/ *S*-Adenosylhomocysteine Nucleosidase	$^{2}T_{3}$	*tg*	0	-
BIG	301A-B	4F3C	1.93	5'-Methylthioadenosine/ *S*-Adenosylhomocysteine Nucleosidase	$^{2}T_{3}$	*tg*	0	-
HCE	301A-B	4F3K	1.85	5'-Methylthioadenosine/ *S*-Adenosylhomocysteine Nucleosidase	^{2}E or $^{2}T_{3}$	*tg*	0	-
BIG	301A	4FFS	1.90	5'-Methylthioadenosine/ *S*-Adenosylhomocysteine Nucleosidase	$^{2}T_{3}$	*tg*	0	-
MTA	300B	4G41	1.45	5'-Methylthioadenosine/ *S*-Adenosylhomocysteine Nucleosidase	$^{4}T_{3}$	*tg*	0	0.95
2WP	301A	4OJT	1.50	5'-Methylthioadenosine/ *S*-Adenosylhomocysteine Nucleosidase	^{2}E	*tg*	0	0.93
SAH	501A	4OY3	1.20	5'-Methylthioadenosine/	$^{4}T_{3}$	*tg*	0	0.95

				S-Adenosylhomocysteine Nucleosidase				
MTA	501A	4P54	1.65	5'-Methylthioadenosine/ *S*-Adenosylhomocysteine Nucleosidase	$^{4}T_{3}$	*tg*	0	0.96
TDI	301A-B	4WKB	1.37	5'-Methylthioadenosine/ *S*-Adenosylhomocysteine Nucleosidase	$^{2}T_{3}$	*tg*	0	-
BIG	301A	4WKC	1.64	5'-Methylthioadenosine/ *S*-Adenosylhomocysteine Nucleosidase	$^{2}T_{3}$	*tg*	0	-
TDI	301A	4WKN	2.00	5'-Methylthioadenosine/ *S*-Adenosylhomocysteine Nucleosidase	$^{2}T_{3}$	*tg*	0	-
GMD	301A	4WKO	1.90	5'-Methylthioadenosine/ *S*-Adenosylhomocysteine Nucleosidase	$^{2}T_{3}$	*tg*	0	-
3QA	301A-D	4WKP	1.58	5'-Methylthioadenosine/ *S*-Adenosylhomocysteine Nucleosidase	^{2}E	*tg*	0	-
TDI	301A-B	4X24	1.50	5'-Methylthioadenosine/ *S*-Adenosylhomocysteine Nucleosidase	^{4}E	*tg*	0	-
4F0	301A	4YML	1.75	5'-Methylthioadenosine/ *S*-Adenosylhomocysteine Nucleosidase	^{N}E	*gt*	0	-
4EH	301A	4YNB	2.00	5'-Methylthioadenosine/ *S*-Adenosylhomocysteine Nucleosidase	$^{2}T_{3}$	*tg*	0	-
SAH	301A-B	5B7N	1.40	5'-Methylthioadenosine/ *S*-Adenosylhomocysteine Nucleosidase	$^{4}T_{3}$	*tg*	0	0.92-0.93
MTA	301A-B	5B7P	1.49	5'-Methylthioadenosine/ *S*-Adenosylhomocysteine Nucleosidase	$^{4}T_{3}$	*tg*	0	0.94
4CT	301A	5KB3	1.40	5'-Methylthioadenosine/	$^{2}T_{3}$	*tg*	0	-

				S-Adenosylhomocysteine Nucleosidase				
C1Y	301A-B	6AYO	1.67	5'-Methylthioadenosine/ *S*-Adenosylhomocysteine Nucleosidase	^{2}E	*gt*	0	-
TDI	301A-B	6AYQ	1.42	5'-Methylthioadenosine/ *S*-Adenosylhomocysteine Nucleosidase	$^{4}T_{3}$ or ^{4}E	*tg*	0	-
BIG	301A-D	6AYR	1.95	5'-Methylthioadenosine/ *S*-Adenosylhomocysteine Nucleosidase	$^{2}T_{3}$	*tg*	0	-
HT6	302A, 303B, 301C, 302D, 301E-F, 302G, 301H	6AYS	1.70	5'-Methylthioadenosine/ *S*-Adenosylhomocysteine Nucleosidase	$^{2}T_{3}$	*tg*	0	-
4EH	301A-D	6AYT	1.85	5'-Methylthioadenosine/ *S*-Adenosylhomocysteine Nucleosidase	^{2}E	*gt*	0	-
OS2	302A, 309B	6DYU	1.60	5'-Methylthioadenosine/ *S*-Adenosylhomocysteine Nucleosidase	$^{2}T_{3}$	*tg*	0	-
OS3	306A, 305B	6DYV	1.62	5'-Methylthioadenosine/ *S*-Adenosylhomocysteine Nucleosidase	$^{2}T_{3}$	*tg*	0	-
OS5	308A, 307B	6DYW	1.45	5'-Methylthioadenosine/ *S*-Adenosylhomocysteine Nucleosidase	$^{2}T_{3}$	*tg*	0	-
OS6	303A, 305B, 303C, 302D	6DYY	1.61	5'-Methylthioadenosine/ *S*-Adenosylhomocysteine Nucleosidase	$^{2}T_{3}$	*tg*	0	-

Table S9. Raw data collected for MTAPs

Ligand ID	Ligand Residue	PDB ID	Res. (Å)	NP Family	Enzyme Function	Ring Conformation	Side Chain Conformation	H-bonding to O5	RSCC
FMC	1330A	1SD1	2.03	NP-I	5'-Methylthioadenosine Phosphorylase	^{4}E	*gt*	0	0.90
MTH	285A	1SD2	2.10	NP-I	5'-Methylthioadenosine Phosphorylase	^{4}E	*gt*	0	0.92
MTA	4003A, 4006B, 4009C, 4012D, 4015E, 4018F, 4021G, 4024H, 4027I, 4030J, 4033K, 4036L	2A8Y	1.45	NP-I	5'-Methylthioadenosine Phosphorylase	^{O}E	*gt*	0	0.91-0.93
4CT	284A	3OZC	1.93	NP-I	5'-Methylthioadenosine Phosphorylase	$^{2}T_{N}$	*gt*	0	-
4CT	284A-B	3OZD	2.10	NP-I	5'-Methylthioadenosine Phosphorylase	$^{2}T_{N}$ or E_{N}	*gt*	0	-
MTA	901A-F	3T94	1.45	NP-I	5'-Methylthioadenosine Phosphorylase	$^{O}T_{1}$	*gt*	0	0.92-0.93
TBN	301A-F	4L5A	2.30	NP-I	5'-Methylthioadenosine Phosphorylase	$^{2}T_{3}$ or $^{4}T_{3}$ or ^{4}E	*gt*	Gln289 (=O, NH) or 0	0.83-0.90
5S7	303A	5EUB	1.81	NP-I	5'-Methylthioadenosine Phosphorylase	$^{2}T_{1}$	*gt*	0	0.95
MTA	301A-C	5F76	1.95	NP-I	5'-Methylthioadenosine Phosphorylase	^{4}E or $^{O}T_{1}$	*gt*	0	0.85-0.91
TBN	301A-C	5F7X	1.77	NP-I	5'-Methylthioadenosine Phosphorylase	^{4}E	*gt*	0	0.85-0.86
BIG	301A-C	5TC5	1.96	NP-I	5'-Methylthioadenosine Phosphorylase	$^{2}T_{N}$ or E_{N}	*gt*	0	-

7A6	301A	5TC6	1.48	NP-I	5’-Methylthioadenosine Phosphorylase	$^{4}T_{N}$	*gt*	0	-
MTH	304A	5TC7	1.75	NP-I	5’-Methylthioadenosine Phosphorylase	^{4}E	*gt*	0	0.94
TDI	301A	5TC8	1.80	NP-I	5’-Methylthioadenosine Phosphorylase	^{2}E	*gt*	0	-
OS2	307A	6DYZ	1.62	NP-I	5’-Methylthioadenosine Phosphorylase	^{2}E	*gt*	0	-
OS3	307A	6DZ0	1.62	NP-I	5’-Methylthioadenosine Phosphorylase	^{2}E	*gt*	0	-
OS5	307A, 303B, 308C	6DZ2	1.99	NP-I	5’-Methylthioadenosine Phosphorylase	E_{N}	*gt*	0	-
OS6	306A, 303B, 307C	6DZ3	1.91	NP-I	5’-Methylthioadenosine Phosphorylase	E_{N}	*gt*	0	-
MTA	401A	8WS2	1.22	NP-I	5’-Methylthioadenosine Phosphorylase	^{O}E	*gt*	0	0.92
MTA	401A	9JD2	1.62	NP-I	5’-Methylthioadenosine Phosphorylase	^{O}E	*gt*	0	0.95
MTA	303A	9JHV	1.65	NP-I	5’-Methylthioadenosine Phosphorylase	^{O}E	*gt*	0	0.95
